# Patterns of Cave Biodiversity and Endemism in the Appalachians and Interior Plateau of Tennessee, USA

**DOI:** 10.1371/journal.pone.0064177

**Published:** 2013-05-22

**Authors:** Matthew L. Niemiller, Kirk S. Zigler

**Affiliations:** 1 Department of Ecology and Evolutionary Biology, Yale University, New Haven, Connecticut, United States of America; 2 Department of Biology, University of the South, Sewanee, Tennessee, United States of America; Ecole Normale Supérieure de Lyon, France

## Abstract

Using species distribution data, we developed a georeferenced database of troglobionts (cave-obligate species) in Tennessee to examine spatial patterns of species richness and endemism, including >2000 records for 200 described species. Forty aquatic troglobionts (stygobionts) and 160 terrestrial troglobionts are known from caves in Tennessee, the latter having the greatest diversity of any state in the United States. Endemism was high, with 25% of terrestrial troglobionts (40 species) and 20% of stygobionts (eight species) known from just a single cave and nearly two-thirds of all troglobionts (130 species) known from five or fewer caves. Species richness and endemism were greatest in the Interior Plateau (IP) and Southwestern Appalachians (SWA) ecoregions, which were twice as diverse as the Ridge and Valley (RV). Troglobiont species assemblages were most similar between the IP and SWA, which shared 59 species, whereas the RV cave fauna was largely distinct. We identified a hotspot of cave biodiversity with a center along the escarpment of the Cumberland Plateau in south-central Tennessee defined by both species richness and endemism that is contiguous with a previously defined hotspot in northeastern Alabama. Nearly half (91 species) of Tennessee’s troglobiont diversity occurs in this region where several cave systems contain ten or more troglobionts, including one with 23 species. In addition, we identified distinct troglobiont communities across the state. These communities corresponded to hydrological boundaries and likely reflect past or current connectivity between subterranean habitats within and barriers between hydrological basins. Although diverse, Tennessee’s subterranean fauna remains poorly studied and many additional species await discovery and description. We identified several undersampled regions and outlined conservation and management priorities to improve our knowledge and aid in protection of the subterranean biodiversity in Tennessee.

## Introduction

Caves and similar habitats are among the most unforgiving environments on the planet. Nonetheless, a taxonomically diverse fauna has been documented from subterranean habitats. For example, more than 1,138 cave-restricted species and subspecies from 112 families and 239 genera have been described in the United States alone [1]. Nearly all of these cave-obligate species (troglobionts) have developed conspicuous regressive and constructive traits uniquely associated with life in perpetual darkness and generally limited food resources, such as loss and reduction of eyes and pigmentation, elongation of appendages, increased longevity, and enhancement of nonvisual sensory modalities [2].

Subterranean biodiversity has been documented for many taxa in the United States [1], [3]–[8], as well as for smaller spatial scales, including the compilation of several state and regional faunal lists [9]–[16]. Of the more than 50,000 caves reported in the United States, nearly 20% occur in Tennessee. Two of the most cave-rich karst regions in the nation, the Interior Low Plateau and the Appalachians, cover much of Tennessee [1], [8], and Tennessee lies just to the north of the hypothesized mid-latitude biodiversity ridge in terrestrial cave fauna in North America [17]. Considerable biospeleological research has been conducted for more than a century in the state [18]–[22]; and references listed in Text S1 and the number of cave-restricted species described from Tennessee has steadily increased during this time. Peck [6] compiled a genus-level summary of obligate subterranean fauna in the United States that included 33 genera of terrestrial troglobionts and 8 genera of stygobionts in Tennessee. The most recent species list for Tennessee is primarily derived from Culver et al.’s [8] study and includes 126 terrestrial troglobionts and 44 stygobionts, ranking second (at 170 species) behind Texas (201 species) for the most obligate subterranean species in the United States [1].

Since Culver et al.’s [8] study, there has been an increase in cave-related research (see references in Text S1) that has dramatically improved our knowledge of the diversity and distribution of the obligate subterranean fauna in Tennessee, including biological inventories and surveys [23]–[28], taxonomic revisions and descriptions of new species [29]–[35], and phylogeographic studies [36]–[42]. Despite the large number of studies on cave-obligate species, spatial patterns of species richness and endemism have not been examined at a local scale in the state. Moreover, most caves in Tennessee are privately owned and afforded little protection. Less than 8% of land is protected in Tennessee (Protected Areas Database of the United States, available online at http://gapanalysis.usgs.gov/padus) and much of this protected area is comprised of landholdings that do not include cave-rich ecoregions. Consequently, there is a need to document local and regional centers of subterranean biodiversity to assist in setting conservation priorities and guiding management decisions for troglobionts in Tennessee.

As a first step toward prioritizing areas for conservation and future research, we compiled all available distributional data for troglobionts to create a georeferenced database of obligate subterranean biota in Tennessee. Using this database, we (1) identified and mapped areas of species richness and endemism for troglobites and stygobites at a local scale in Tennessee; (2) examined the taxonomic composition of local and regional species assemblages; (3) defined cave biogeographic regions based on similarity of cave communities; and (4) evaluated gaps in our knowledge of Tennessee’s cave biodiversity. In addition, we examine potential processes underlying observed patterns of biodiversity and endemism as well as the implications of these patterns for conservation and management of cave faunas.

## Materials and Methods

### Ethics Statement

Biological surveys that generated data not included in other published studies were conducted in accordance with protocols approved by the Institutional Animal Care and Use Committee at the University of Tennessee-Knoxville (protocol no. 1589–0507) and Yale University (protocol no. 2012–10681), and under authorization of the Tennessee Wildlife Resources Agency (permit nos. 1585 and 1605) and the Tennessee Department of Environment and Conservation (permit no. 2011-005). Efforts were made to minimize the number of specimens collected for proper identification and to minimize habitat disturbance during biological surveys.

### Study Area

**Figure 1:**
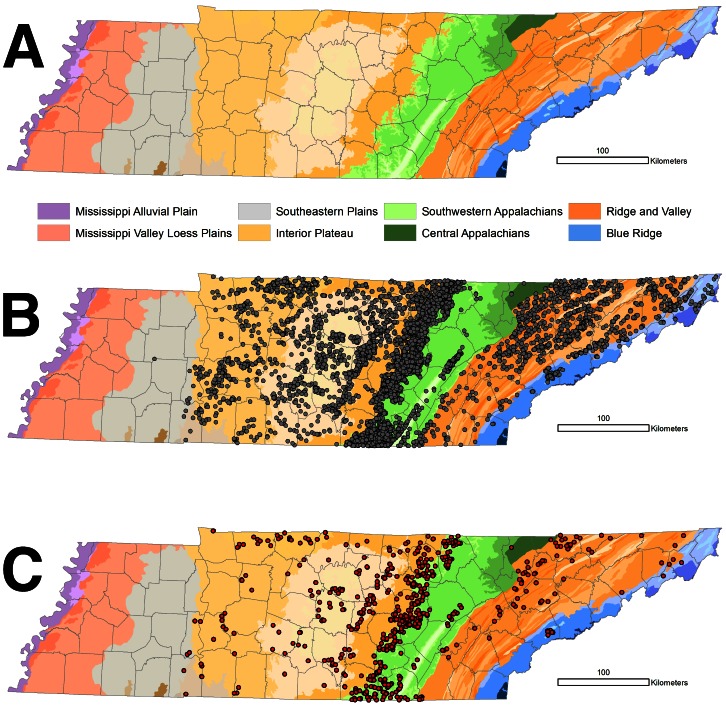
Ecoregions and cave distribution in Tennessee. (a) Ecoregions of Tennessee [following 79]. The eight Level III ecoregions are labeled and Level IV subdivisions of these ecoregions are individually colored with color themes (e.g., oranges, blues, and greens) corresponding to the Level III ecoregions. (b) Distribution of 9517 georeferenced caves and (c) 661 caves with at least one troglobiont recorded in Tennessee overlaid onto ecoregions. Cave and karst regions occur in six Level III ecoregions with the greatest density of caves in the Interior Plateau, Southwestern Appalachians, and Ridge and Valley. The majority of caves occur in exposed geological strata along escarpments marking the transition between ecoregions. County boundaries are also highlighted.

Two major karst biogeographic regions, the Interior Plateau and the Appalachians, occur in Tennessee [1], [8]. More than 9500 caves have been reported from the state, with the greatest density occurring along the western margin of the Cumberland Plateau (Fig. 1). Caves have been reported from six of the eight Level III ecoregions recognized in Tennessee. These ecoregions are generally orientated north-south across the state from west to east (Fig. 1). Caves and karst are most extensively developed in the Interior Plateau (IP), Southwestern Appalachians (SWA), and Ridge and Valley (RV) ecoregions in the central and eastern part of the state (Table 1).

**Table 1:** Caves, cave density, and caves with troglobiont records (“sampled caves”) by ecoregion in Tennessee.

Ecoregion	Area (km^2^)	No. of caves	caves/100 km^2^	No. of sampled caves	Pct. of sampled caves
**Southeastern Plains**	**13,318**	**14**	**0.1**	**4**	**28.6%**
**Interior Plateau**	**40,724**	**2834**	**7.0**	**278**	**9.8%**
Western Highland Rim	15,236	424	2.8	29	6.8%
Western Pennyroyal Karst Plain	2,137	230	10.8	24	10.4%
Inner Nashville Basin	4,324	368	8.5	29	7.9%
Outer Nashville Basin	11,468	787	6.9	85	10.8%
Eastern Highland Rim	7,558	1,025	13.6	111	10.8%
**Southwestern Appalachians**	**12,497**	**5011**	**40.1**	**289**	**5.8%**
Cumberland Plateau	8,235	107	1.3	6	5.6%
Plateau Escarpment	3,607	4791	132.8	260	5.4%
Sequatchie Valley	651	113	17.4	23	20.4%
**Central Appalachians**	**2,302**	**18**	**0.8**	**2**	**11.1%**
**Ridge and Valley**	**19,600**	**1469**	**7.5**	**75**	**5.1%**
**Blue Ridge Mountains**	**6,379**	**171**	**2.7**	**13**	**7.6%**
**Total**	**94,820**	**9517**	**10.0**	**661**	**6.9%**

The six Level III ecoregions that contain caves are shown in bold. Also shown are Level IV ecoregion subdivisions of the Interior Plateau and the Southwestern Appalachians.

The IP in Tennessee is divided into five Level IV ecoregions, each with significant areas of caves and karst: Western Pennyroyal Karst, Western Highland Rim, Eastern Highland Rim, Outer Nashville Basin, and Inner Nashville Basin (Table 1). The Highland Rim encircles the oval-shaped Nashville Basin and is 150–180 m higher in elevation. Much of the IP in Tennessee is underlain by soluble carbonate strata and exhibits moderately to well-developed karst topography [43]. Three major karst terranes occur in the IP of Tennessee, including the Highland Rim Karst, Nashville Basin Karst, and Cumberland Plateau Karst, the last of which also includes the Plateau Escarpment ecoregion of the SWA [44], [45]. The Highland Rim Karst and Cumberland Plateau Karst are developed in Early to Middle Mississippian-age strata, whereas the Nashville Basin Karst is developed in Early to Middle Ordovician-age limestones. Most caves developed in the IP occur in exposed strata along escarpments, marking the boundaries between ecoregions.

To the east of the IP is the SWA (Fig. 1a), which is subdivided into three Level IV ecoregions: the Plateau Escarpment, the Cumberland Plateau, and the Sequatchie Valley (Table 1). The major topographic feature of the SWA is the Cumberland Plateau, an elevated upland (550–610 m above sea level (ASL)) bounded to the east by the RV ecoregion and to the west by the Eastern Highland Rim of the IP (275–350 m ASL). The Cumberland Plateau is capped by the Pennsylvanian-aged sandstone overlaying Mississippian-aged limestones. This hydrogeological setting is optimal for cave development [46], [47]. Almost 180 million years of differential lowering between Cumberland Plateau and Eastern Highland Rim has created a highly-dissected, eastward-retreating escarpment along the western margin of the Cumberland Plateau [47]. Cave density in Tennessee peaks along the Plateau Escarpment ecoregion (Table 1, Fig. 1), with the oldest cave passages dated to 5.7 Mya [47]. Cave development also is prominent within and along the margins of the Sequatchie Valley, which is an open, rolling valley averaging 6.4 km wide and extending 240 km from Cumberland County in Tennessee into northwest Alabama. This ecoregion is associated with an anticline where erosion has formed a deep valley nearly 300 m lower in elevation than the surrounding Cumberland Plateau.

The RV consists of a series of mainly parallel ridges and valleys that generally run from southwest to northeast between the SWA and Central Appalachians to the west and the Blue Ridge Mountains to the east. Ordovician-age limestones and dolomites characterize this ecoregion, with elevations ranging 210–610 m ASL and local relief up to 210 m. Rock layers in this ecoregion have been significantly faulted and folded due to past tectonic events associated with the uplift of the Appalachian Mountains.

### Database Compilation

We created a database of distributional records for all formally described species restricted to caves and other associated subterranean habitats (e.g., phreatic waters) in Tennessee. Our database also included records for taxa considered undescribed and new to science in the literature or via personal communication with taxonomic experts. We followed the definition of Sket [48] with ‘troglobiont’ referring to any species strictly bound to subterranean habitats. In practice, species were considered troglobionts if they had few or no records from surface habitats, were described as cave obligates by previous authors, or exhibited troglomorphic features, such as the reduction or loss of eyes, little to no pigmentation, and elongation of appendages [2]. Troglobionts were further classified based on habitat, as terrestrial troglobionts that occur in terrestrial subterranean habitats and as stygobionts that occur in aquatic subterranean habitats.

We excluded species considered as eutroglophiles, subtroglophiles, and trogloxenes (following [48]) that were not obligately associated with subterranean habitats. Such species were identified on the basis of having several records from surface habitats or having been classified as troglophiles, trogloxenes, or accidentals by previous authors. We excluded non-troglobionts from the current study because (1) many species occasionally enter caves and their degree of cave association is often difficult to determine, (2) cave studies and surveys report non-troglobionts to varying degrees, and (3) troglobionts are a coherent ecological grouping of species that are restricted to subterranean habitats and usually exhibit distinct morphological features aiding in their ecological classification compared to non-troglobionts. Information on non-troglobiont cave biodiversity in Tennessee can be found in several papers [10], [20], [24], [26], [39], [49], [50].

Distributional records were compiled from several sources, including relevant scientific literature, existing biodiversity databases, and personal records. Literature records were assembled from peer-reviewed journals, books, theses and dissertations, government reports, and caving organization newsletters. This included keyword searches of ISI Web of Science and Google Scholar and examining in detail all references cited in the resulting articles. A full list of references is provided in Text S1. We also obtained records from biodiversity databases maintained by the Tennessee Natural Heritage Inventory Program (TNHP), the Tennessee Chapter of The Nature Conservancy (TNC), and the Tennessee Cave Survey (TCS). The database also was supplemented with reliable unpublished distributional records maintained by several taxonomic specialists, as well as new records resulting from our own biospeleological surveys. Biological surveys consisted primarily of visual encounter surveys of terrestrial and aquatic cave habitats as well as trapping for terrestrial invertebrates (i.e., baited pitfall traps) and aquatic invertebrates (i.e., baited funnel traps).

All distributional records from caves were incorporated into an ArcGIS (v.10) database along with spatial information (geographic coordinates, ecoregion, county, etc.). We attempted to georeference each distribution record using a database of caves in Tennessee maintained by TCS. Some 9705 caves have been recorded in Tennessee, with 9517 caves that have been reliably georeferenced and included in our study (Fig. 1). The TCS requires caves to have a horizontal length of 50′, a total vertical extent of 40′, or a 30′ pit to be included in their database. In addition, we cross-referenced our biological database with the databases maintained by TNHP, TNC, TCS, and a U.S. cave biodiversity database compiled by Culver et al. [8], which is available online at http://www.karstwaters.org. For several reasons, some records were excluded, including taxonomic revision leading to synonymy of species, records that were questionable or revised in the literature, typographic errors, duplicate records, erroneous locality information, and improper classification as troglobionts in the literature based on the criteria mentioned previously.

Troglobiont distributional records in the database were translated into a presence-absence matrix, in which each cave locality represented a row in the matrix and each column represented a single species. This matrix was used in analyses of taxonomic diversity and species richness. The list of troglobionts and the presence-absence matrix are available in Table S1 and Dataset S1, respectively. To protect sensitive cave habitats and species, as well as copyrighted data of the TCS, cave locations are not included. Please contact the authors or appropriate organizations (i.e., TNHP, TNC, and TCS) for data requests.

### Spatial Patterns of Biodiversity

We examined spatial patterns of subterranean biodiversity by generating a grid-based distribution map of species richness and endemism in ArcGIS. We mapped distributional patterns by overlaying a grid of 20×20 km cells (400 km^2^) onto a base map of Tennessee. A total of 321 grid cells covered the entire state, with 215 of these cells containing one or more caves. Each georeferenced record in the database was then assigned to a cell of this grid. Distribution maps of species richness and endemism were produced by counting the number of species and endemics (see below) present in the 321 cells of the grid coverage. Some previous studies examined cave biodiversity at the county level (e.g., [8]). To facilitate comparison to these studies, we also mapped species richness and single-site endemism at the county level.

### Sampling Effort and Gap Analyses

Determining to what extent species richness within a given 20×20 km grid cell reflects true diversity or sampling effort is difficult from our dataset alone, as we did not include distributional data of non-troglobiotic fauna. Therefore, we employed approaches at several scales – across the state, by Level III and Level IV ecoregions, and by 20×20 km cell – to identify and evaluate potential gaps. First, we used Spearman’s Rank Correlation test to determine if a correlation between the total number of caves and the number of sampled caves with at least one troglobiont in a grid cell existed. Cells that lacked caves were excluded. A strong correlation over the entire study region would suggest that there was not a significant bias in geographic extent of sampling effort. Second, we noted the percentage of caves with troglobiont records (“sampled caves”) across ecoregions to identify ecoregions that had been sampled at a higher or lower rate. Third, we used two methods to identify ‘undersampled’ cells. We first identified cells with the greatest negative standardized residuals from the best-fit line relating caves/cell and sampled caves/cell. We also used a threshold approach to identify all cells where <3% of caves had been sampled to identify cells that had been sampled at a much lower rate than the 6.9% of caves statewide that had been sampled. Cells with fewer than 10 caves were excluded from the threshold analysis. For these and all other analyses, all caves were counted equally; we did not consider cave length or depth.

### Taxonomic Distinctness

Taxonomic distinctness was calculated using the metric average taxonomic distinctness (Δ^+^), which is the mean of distances through a classification tree for all pairs of species in a sample [51]. Higher Δ^+^ values imply a more taxonomically diverse species assemblage, whereas lower values imply lower taxonomic diversity. This metric was calculated for each cave as a sample using the *vegan* package v2.0.4 [52] in R v2.15.1 [53]. Because a phylogeny is not available for all subterranean organisms, the Linnean hierarchical levels (i.e., phylum, class, order, family, genus, species) were translated into an input classification tree following the Integrated Taxonomic Information System (ITIS) taxonomically structured species database [54]. Δ^+^ was scaled to a maximum 100 for the most taxonomically unrelated species. This metric was calculated for all troglobionts, terrestrial troglobionts, and stygobionts overall and for each ecoregion. We tested for differences in Δ^+^ between the three main ecoregions (IP, SWA, and RV) using a Kruskal-Wallis rank sum test after examination of normality plots. A post-hoc multiple comparison test was used to determine which pairwise comparisons were different using the *pgirmess* package v1.5.4 in R.

### Observed and Estimated Species Richness

Species accumulation curves were constructed in the *vegan* package by randomly subsampling caves without replacement [55]. We also estimated extrapolated species richness from the observed samples (caves) using three non-parametric incidence-based estimators, Chao2 [56], [57], first-order jack-knife [58], and bootstrap richness estimator [59]. We chose to employ a variety of richness estimators because no single estimator has been shown to be best suited across all situations and taxa [60]. Species accumulation curves were constructed for all troglobionts, terrestrial troglobionts, and stygobionts overall and for each ecoregion. We also tested for differences in observed species richness among ecoregions using a Kruskal-Wallis rank sum test.

### Endemism

We examined patterns of endemism at two scales. First, we considered the number of species that occur at only a single cave (i.e., single-site endemics). We also considered the number of species present in only one grid cell of the sampling grid as a measure of local endemism (i.e., single-cell endemics). We tested for a correlation between endemism and species richness for both sites and grid cells using Spearman’s Rank Correlation test. Cells that lacked caves were excluded.

### Subterranean Community Composition

We used multivariate analyses to identify caves that had similar troglobiont communities and to identify biogeographic breaks in troglobiont communities. We used the Multivariate Statistical Package v3.1 [61] to conduct Principal Components Analysis (PCA) and Detrended Correspondence Analysis (DCA) on a presence/absence matrix of troglobionts, generating a scatterplot where each cave was represented by a single point. We included caves with eight or more known troglobionts (N = 58 from 16 counties), after excluding three caves (TCB9 in Claiborne County, TMN26 in Marion County and TBD1 in Bledsoe County) that preliminary analyses identified as extreme outliers. TCB9 was the only cave in the RV with eight or more troglobionts, and TMN26 and TBD1 were the only caves on the eastern escarpment of the Cumberland Plateau with eight or more troglobionts. To interpret the clustering we observed in the PCA and DCA, we looked for correspondence between those clusters and four regional boundaries: counties, ecoregions, 20 km×20 km cells, and U.S. Geological Survey-defined subbasins (HUC8).

## Results

### Database Overview and Sampling Effort

We compiled 2287 records of described troglobionts from Tennessee, of which 96% (1976 records) could be georeferenced. Another 18 records were from eight localities that were not in the cave database because these localities failed to meet the minimize length or depth requirements to be considered a cave by the TCS. Sixty-three records could not be confidently assigned to a known cave in the database. Our working dataset included 1976 records from 661 caves (Fig. 1c), representing 196 species. We also compiled 147 records for taxa reported as “new” or “undescribed” species and 83 records that could not be reliably identified to the species level. Of these 230 records, 98% could be confidently assigned to a cave in the TCS cave database. However, these records, as well as those that could not be georeferenced, were excluded from subsequent analyses.

Just 6.9% of all caves in Tennessee have records of troglobionts. Most sampled caves were concentrated in the Plateau Escarpment of the SWA and in the IP (Table 1). The cumulative number of described troglobionts has increased with time (Fig. S1). Since 1950, the number of species has increased by 270% from 54 species to 200 species at present. However, the rate of new species reported from Tennessee has slowed since 1980 despite an increase in the number of studies and publications.

### Taxonomic Diversity

The compiled database contained records for 55 genera and 200 described species (including three subspecies), which included four phyla, ten classes, and 22 orders of invertebrates as well as two classes and orders of vertebrates (Table 2). Terrestrial troglobionts accounted for 80% (160 species) of all troglobionts, whereas stygobionts accounted for 20% (40 species). Coleoptera (beetles; ten genera and 72 species), chordeumatid millipedes (two genera and 29 species), and pseudoscorpions (six genera and 18 species) were the most diverse terrestrial orders, comprising 74.4% of all terrestrial troglobionts. Amphipods (three genera and 12 species) and isopods (two genera and ten species) were the most diverse aquatic groups.

**Table 2:** Taxonomic diversity of cave-obligate species in Tennessee, including number of genera, number of described species, number of single-cave endemics, number of single 20×20 km cell endemics, and number of occurrence records.

Taxon	No. genera	No. of describedspecies	No. of single-cave endemics	No. of single-cell endemics	No. of records
Annelida					
Clitellata					
Branchiobdellida	1	2	2	2	2
Lumbriculida	1	1	1	1	1
Platyhelminthes					
Turbellaria					
Tricladida	1	3	0	0	30
Mollusca					
Gastropoda					
Basommatophora	1	1	0	0	10
Stylommatophora	2	3	0	0	21
Arthropoda					
Arachnida					
Acari	1	1	0	0	3
Araneae	6	14	3	3	201
Opiliones	1	1	0	0	18
Pseudoscorpiones	6	18	9	10	101
Diplopoda					
Callipodida	1	2	0	0	55
Chordeumatida	2	29	2	11	178
Julida	1	1	0	0	8
Polydesmida	1	2	1	1	7
Malacostraca					
Amphipoda	3	12	3	3	115
Decapoda	2	5	0	0	267
Isopoda	3	11	2	3	223
Maxillopoda					
Cyclopoida	2	3	0	0	12
Ostracoda					
Podocopida	2	2	0	0	14
Hexapoda					
Collembola	4	10	0	0	118
Diplura	1	3	0	0	61
Insecta					
Coleoptera	10	72	25	29	336
Diptera	1	1	0	0	118
Chordata					
Actinopterygii					
Percopsiformes	1	1	0	0	109
Amphibia					
Caudata	1	2	0	0	49
Total	55	200	48	63	2057

This list does not include undescribed species or records that were not identified to the species level.

**Figure 2:**
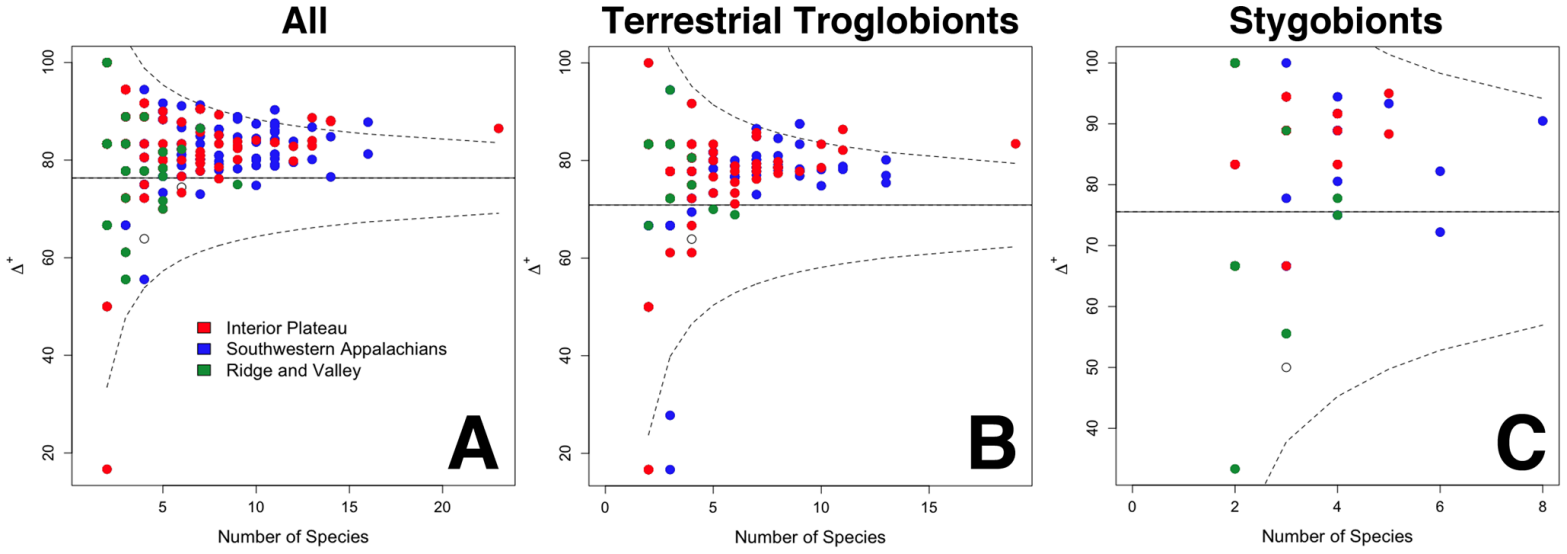
Average taxonomic distinctness (Δ^+^) for caves of Tennessee: (a) all troglobionts, (b) terrestrial troglobionts only, and (c) stygobionts only. Each point represents a cave. Ecoregions are color-coded. The solid line is the simulated mean value and the funnel curve shows the 95% confident limits of expected values.

Most caves had Δ^+^ values higher than the simulated mean but within 95% confidence limits (Fig. 2a). Nine caves (four in the IP and five in the SWA) had higher than expected Δ^+^ for all troglobionts, whereas no caves in the RV had higher or lower than expected Δ^+^. A single cave (TWR10 in the IP) had lower than expected Δ^+^. Three caves had higher than expected Δ^+^ for all troglobionts and one other cave in the IP had higher than expected Δ^+^ for terrestrial troglobionts (Fig. 2b) but not stygobionts (Fig. 2c). Four caves had lower than expected Δ^+^ for terrestrial troglobionts, but not for stygobionts. Average taxonomic distinctness of troglobionts varied among ecoregions, with the highest values in the IP and SWA (Table 3). However, differences were only significant between the IP and RV. All ecoregions had similar values of Δ^+^ when considering terrestrial troglobionts only, whereas the IP and SWA had similar values of Δ^+^ for stygobionts, which were both significantly higher than the RV (Table 3).

**Table 3:** Mean ± SD of taxonomic distinctness (Δ^+^) and species richness (S_obs_) of subterranean biodiversity in Tennessee per Level III ecoregion for all troglobionts, terrestrial troglobionts, and stygobionts.

	Interior Plateau	Southwestern Appalachians	Ridge & Valley	Inter-ecoregion differences	Significant pairwise comparisons
**Taxonomic distinctness**					
All	83.9±10.4	83.0±8.1	79.2±11.1	H = 10.84, df = 2, P<0.01	RV vs. IP
Terrestrial troglobionts	77.9±12.6	78.5±9.0	79.1±7.0	H = 2.50, df = 2, P>0.05	none
Stygobionts	85.5±13.5	85.5±13.0	73.7±17.8	H = 8.93, df = 2, P<0.05	RV vs. IP, RV vs. SWA
**Species richness**					
All	2.9±3.0	3.3±3.4	2.3±1.6	H = 0.31, df = 2, P>0.05	none
Terrestrial troglobionts	1.8±2.4	2.0±2.9	1.2±1.3	H = 1.35, df = 2, P>0.05	none
Stygobionts	1.1±1.1	1.3±1.2	1.1±1.0	H = 6.02, df = 2, P>0.05	none

### Species Richness

**Figure 3:**
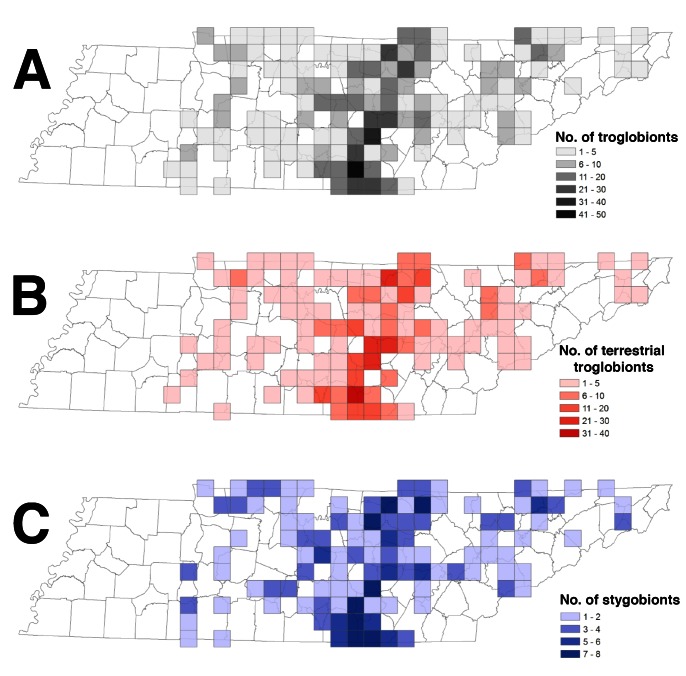
Spatial patterns of species richness in 20×20 km grid cells distributed across Tennessee, including (a) 196 cave-obligate species with mappable occurrence records, (b) terrestrial troglobionts only, and (c) stygobionts only.

Species richness was greatest along the escarpments of the Cumberland Plateau marking the transition from the SWA into the IP (Fig. 3a), particularly the southern section where greatest richness occurred in northeastern Franklin, southwestern Grundy, and northwestern Marion counties (Fig. S2). In contrast, troglobiont species richness was greatest in the northern RV in Claiborne and Hancock counties. Species richness differed among the three major cave-containing ecoregions, with greatest species richness in the IP and SWA and lowest species richness in the RV (Table 4). Among Level IV ecoregions, greatest species richness was observed in the Plateau Escarpment of the SWA (98 species) followed by the adjacent Eastern Highland Rim (66 species) and Outer Nashville Basin (53 species) of the IP to the west (Table 4; Fig. S3). Observed terrestrial troglobiont richness followed an identical pattern with overall troglobiont richness, with greatest species richness observed in the IP and SWA, specifically within the Plateau Escarpment ecoregion (79 species) of the SWA (Table 4, Fig. 3b). Observed stygobiont richness also was greatest in the IP and SWA, with greatest species richness in the Plateau Escarpment (Table 4, Fig. 3c).

**Table 4:** Sampled caves, troglobionts (Obs.), single-cave endemics (End.), and estimated species richness by ecoregion in Tennessee.

				Overall			Terrestrial Troglobionts				Stygobionts			
Ecoregion	Caves	Obs.	End.	Chao	JK1	BS	Obs.	Chao	JK1	BS	Obs.	Chao	JK1	BS
**Southeastern Plains**	**4**	**3**	**0**	**5±4**	**5±1**	**4±1**	**1**	**1±0**	**2±1**	**1±0**	**2**	**3±1**	**3±1**	**2±0**
**Interior Plateau**	**278**	**116**	**19**	**179±27**	**158±8**	**134±5**	**98**	**155±26**	**135±8**	**114±4**	**18**	**24±8**	**23±2**	**20±1**
Western Highland Rim	29	29	3	41±9	42±5	35±3	22	34±10	33±4	27±2	7	8±2	9±1	8±1
Western Pennyroyal Karst Plain	24	15	0	15±1	17±1	17±2	11	11±1	13±1	13±2	4	8±0	4±0	8±0
Inner Nashville Basin	29	16	0	25±10	22±4	18±2	10	23±17	15±3	12±2	6	7±1	7±1	7±1
Outer Nashville Basin	85	53	11	78±14	74±6	62±3	43	61±12	60±5	51±3	10	18±12	14±2	12±1
Eastern Highland Rim	111	66	5	118±31	91±7	76±4	52	82±19	71±6	60±4	14	14±0	20±3	16±1
**Southwestern Appalachians**	**289**	**102**	**15**	**134±15**	**133±8**	**116±5**	**79**	**107±15**	**105±7**	**91±4**	**23**	**27±5**	**28±2**	**25±1**
Cumberland Plateau	6	17	0	59±39	28±9	22±5	16	52±33	26±8	20±4	1	1±0	2±1	1±0
Plateau Escarpment	260	98	13	126±13	129±8	112±5	75	97±12	100±7	86±4	23	29±5	29±2	26±2
Sequatchie Valley	23	29	2	43±10	41±7	35±4	20	33±11	30±5	24±2	9	11±3	12±3	10±2
**Central Appalachians**	**2**	**4**	**1**	**4±0**	**6±2**	**5±1**	**3**	**3±0**	**5±1**	**4±1**	**1**	**1±0**	**2±1**	**1±0**
**Ridge and Valley**	**75**	**50**	**10**	**120±40**	**79±7**	**62±3**	**37**	**103±44**	**60±6**	**46±3**	**13**	**22±10**	**19±2**	**15±1**
**Blue Ridge Mountains**	**13**	**9**	**2**	**14±7**	**12±2**	**10±1**	**5**	**6±1**	**6±1**	**5±1**	**4**	**4±0**	**6±1**	**5±1**
Total	661	196	47	286±30	262±10	225±5	158	239±30	215±9	183±5	38	48±8	48±3	42±2

The six Level III ecoregions that contain caves are highlighted in bold. Also shown are Level IV ecoregions (subregions or Level III ecoregions) of the Interior Plateau and Southwestern Appalachians. Observed troglobiont species richness do not sum because some species are present in more than one ecoregion. We also estimated extrapolated species richness from sampled caves using three non-parametric incidence-based estimators, including Chao2 (Chao), first-order jack-knife (JK1), and bootstrap richness estimator (BS). Four troglobionts (including one single-cave endemic) are not included here because their records could not be reliably georeferenced.

Troglobiont species richness averaged 3.0±3.0 species per sampled cave. Thirty-nine caves contained ten or more troglobionts, including three caves with 15 or more species and one cave with 23 species (Table 5). All of these caves were located in either the IP or SWA. Over 47% (313) of caves were represented by a single documented species. Terrestrial troglobiont species richness averaged 1.8±2.5 species per cave. Twelve caves contained ten or more terrestrial troglobiont species with a maximum of 19 species in a single cave in the IP. Stygobiont species richness averaged 1.2±1.1 species per cave. Six caves contained five or more species with a maximum of eight species at a single cave in the SWA. There was a strong association between terrestrial troglobiont species richness and stygobiont species richness (*r = *0.79, *P*<0.001). Observed species richness for all troglobionts, terrestrial troglobionts, and stygobionts did not differ among major ecoregions (Table 3).

**Table 5:** Tennessee caves and cave systems with the greatest number of cave-obligate species.

Cave	County	Ecoregion	TCS No.	No. of species	No. of types
Crystal Cave	Grundy	IP – Eastern Highland Rim	TGD10	23	2
Big Mouth Cave	Grundy	SWA – Plateau Escarpment	TGD2	16	0
Dry Cave	Franklin	SWA – Plateau Escarpment	TFR9	16	2
Tom Pack Cave	Franklin	SWA – Plateau Escarpment	TFR87	14	0
Little Slippery Slit Cave	Overton	IP – Eastern Highland Rim	TOV427	14	0
Trussell Cave	Grundy	IP – Eastern Highland Rim	TGD26	14	1
Cumberland Caverns	Warren	SWA – Plateau Escarpment	TWR7	14	6
McElroy Cave	Van Buren	SWA – Plateau Escarpment	TVB10	13	2
Herring Cave	Rutherford	IP – Inner Nashville Basin	TRU8	13	1
Keith Cave	Franklin	SWA – Plateau Escarpment	TFR14	13	0
Swamp River Cave	Van Buren	IP – Eastern Highland Rim	TVB657	13	0
Skull Cave	Grundy	IP – Eastern Highland Rim	TGD24	13	0
Bunkum Cave	Pickett	IP – Eastern Highland Rim	TPI2	12	3
Caney Hollow Cave	Franklin	IP – Outer Nashville Basin	TFR2	12	1
Grapevine Cave	Franklin	SWA – Cumberland Plateau	TFR423	12	0
Walker Spring Cave	Franklin	IP – Eastern Highland Rim	TFR28	12	0
**Cave system**	**County**	**Ecoregion**	**TCS No.**	**No. of species**	**No. of types**
Crystal/Wonder Cave System	Grundy	IP – Eastern Highland Rim	TGD10, TGD30	24	6
Big Mouth/Big Room Cave System	Grundy	SWA – Plateau Escarpment	TGD2, TGD3, TGD20	20	0
Rumbling Falls Cave System	Van Buren	IP – Eastern Highland Rim	TVB657, TVB588, TVB515, TVB352	17	0

Undescribed species are not included. The unique Tennessee Cave Survey number (TCS No.) for each cave, ecoregion (Level III and Level IV), species richness (No. of species), and the number of species for which each cave/cave system is the type locality (No. of types) are also noted.

**Figure 4:**
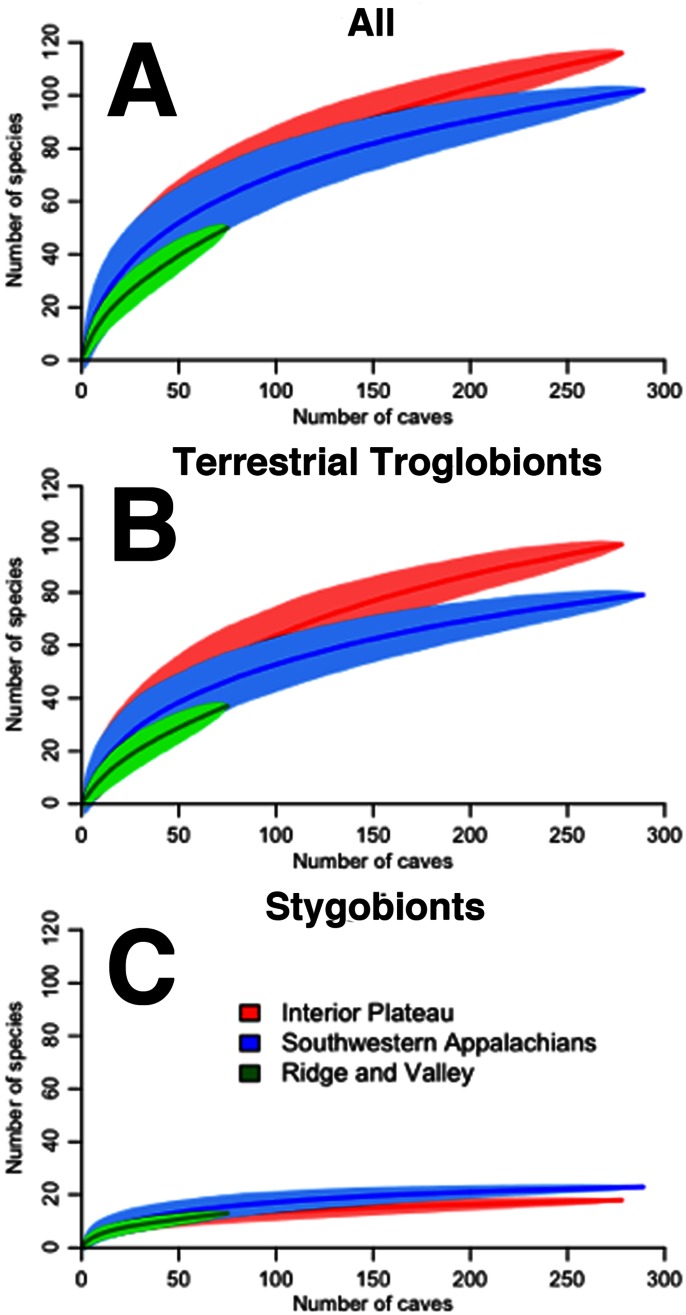
Species accumulation curves for (a) all troglobionts, (b) terrestrial troglobionts, and (c) stygobionts in the three major cave-bearing ecoregions in Tennessee. The shaded area around each line represents the 95% confidence interval.

Observed species richness was highly correlated with sampling effort (number of caves with records in a grid cell; *r* = 0.95, P<0.001), with nearly identical correlation coefficients when considering just terrestrial troglobionts (*r* = 0.88, P<0.001) and just stygobionts (*r* = 0.89, P<0.001), respectively. Species accumulation curves did not approach an asymptote for all troglobionts (Fig. 4a) in each major ecoregion, but this was driven primarily by terrestrial troglobionts (Fig. 4b), as stygobionts did approach an asymptote (Fig. 4c). This suggests that the current level of sampling captured total species richness well for aquatic cave taxa but not for terrestrial species. The three richness estimates showed that the observed species richness of all troglobionts represented at least 69% of estimated species richness, with observed terrestrial troglobiont richness and observed stygobiont richness representing at least 66% and 79% of total estimated richness, respectively (Table 4).

### Endemism

**Figure 5:**
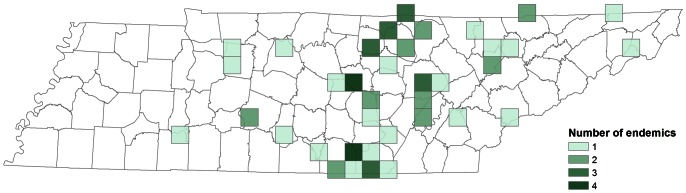
Spatial patterns of endemism in 20×20 km grid cells distributed across Tennessee: number of single-cell troglobionts.

**Figure 6:**
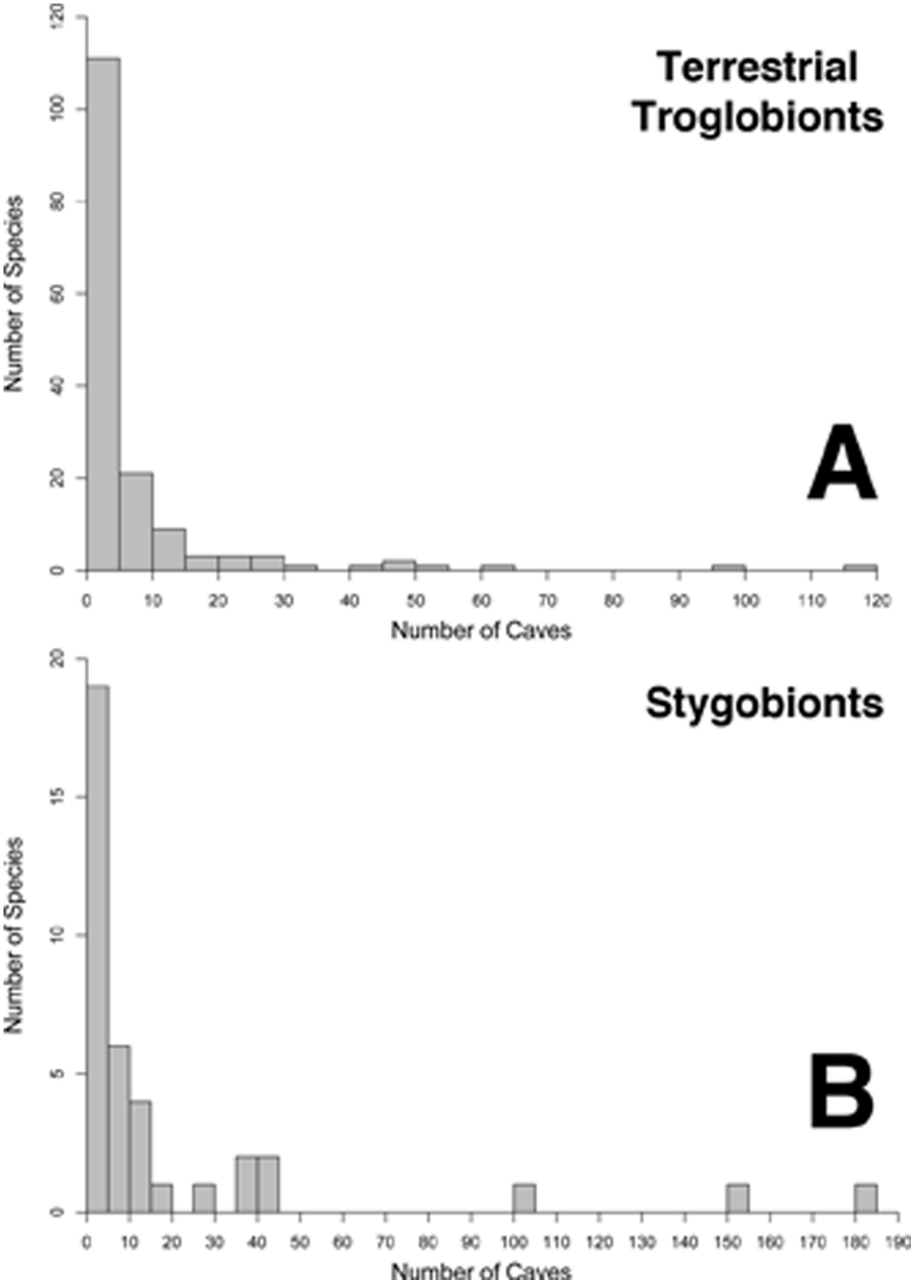
Histogram of cave records in Tennessee for (a) terrestrial troglobionts and (b) stygobionts. Most species of terrestrial troglobionts and stygobionts have been reported from five or fewer caves.

Most troglobionts have small geographic ranges in Tennessee. 31.5% (63 species) of troglobionts were known from a single 20×20 km cell, with 24% (48 species) known from just a single site (Fig. 5). Forty single-site endemics were terrestrial troglobionts, including 25 species of beetles (order Coleoptera) and nine species of pseudoscorpions (order Pseudoscorpiones) (Table 2). Almost two-thirds (130 species) of all troglobionts in Tennessee are known from five or fewer caves, including 111 terrestrial troglobionts (69% of all terrestrial troglobionts) and 19 stygobionts (48% of all stygobionts) (Fig. 6). Only 22 troglobionts (14 terrestrial troglobionts and eight stygobionts) are known from 20 or more caves and just five species (the isopod *Caecidotea bicrenata*, the crayfish *Orconectes australis*, the spider *Phanetta subterranea*, the fly *Spelobia tenebrarum*, and the cavefish *Typhlichthys subterraneus*) have been reported from 100+ caves. Six caves were home to more than one single-site endemic: TMN26 in Marion County had three single-site endemic species, whereas TCB9 (Claiborne County), TMU1 (Maury County), TCY13 (Clay County), TWR7 (Warren County) and TRH2 (Rhea County) each had two single-site endemic species.

Single-cell (*r* = 0.31, *P*<0.001) and single-site endemicity (*r* = 0.23, *P*<0.001) were weakly correlated with the number of caves per grid cell. The number of single-cell endemics (*r* = 0.55, *P*<0.001) and single-site endemics (*r* = 0.48, *P*<0.001) were also positively correlated with species richness per grid cell.

### Gap Analysis

The number of caves sampled was correlated with the total number of caves per grid cell across Tennessee (*r* = 0.67, *P*<0.001). This suggests a reasonably even level of sampling across the state. The percentage of sampled caves ranged 5.1–28.6% within Level III ecoregions that contained caves, and 5.1–9.8% among the three major cave ecoregions (Table 1). Within Level IV ecoregions of the IP and SWA, the percentage of sampled caves ranged 5.4–10.8% with the exception of the Sequatchie Valley, where 20.4% of caves have been sampled (Table 1).

**Figure 7:**
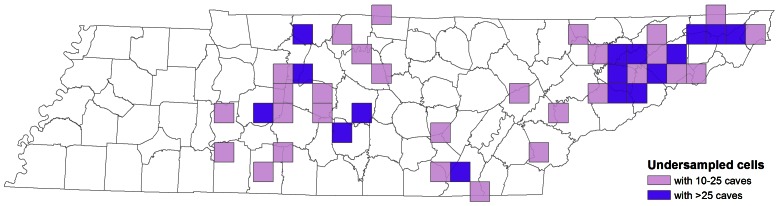
Spatial patterns of sampling gaps in 20×20 km grid cells distributed across Tennessee. All cells where less than 3% of caves have troglobiont records are highlighted.

On a smaller scale, we identified grid cells that were undersampled by two different methods–using residuals and using a threshold. Both methods identified similar groups of cells (74% overlap between the two approaches), but the threshold method included cells with fewer caves and omitted some cells with hundreds of caves that had been sampled at >3%. We preferred the threshold method as it emphasized cells that had not been sampled at all, even when the cell contained relatively few caves. The threshold method identified 16 cells that contain more than 25 documented caves where <3% had been sampled. These cells were concentrated in northeast Tennessee in the RV but also scattered across the IP (Fig. 7). We identified 29 grid cells that contain 10–25 documented caves and have not had a single cave sampled. These cells were also concentrated in the northeast RV, with other undersampled cells scattered across the southern RV and IP (Fig. 7).

### Troglobiont Communities

**Figure 8:**
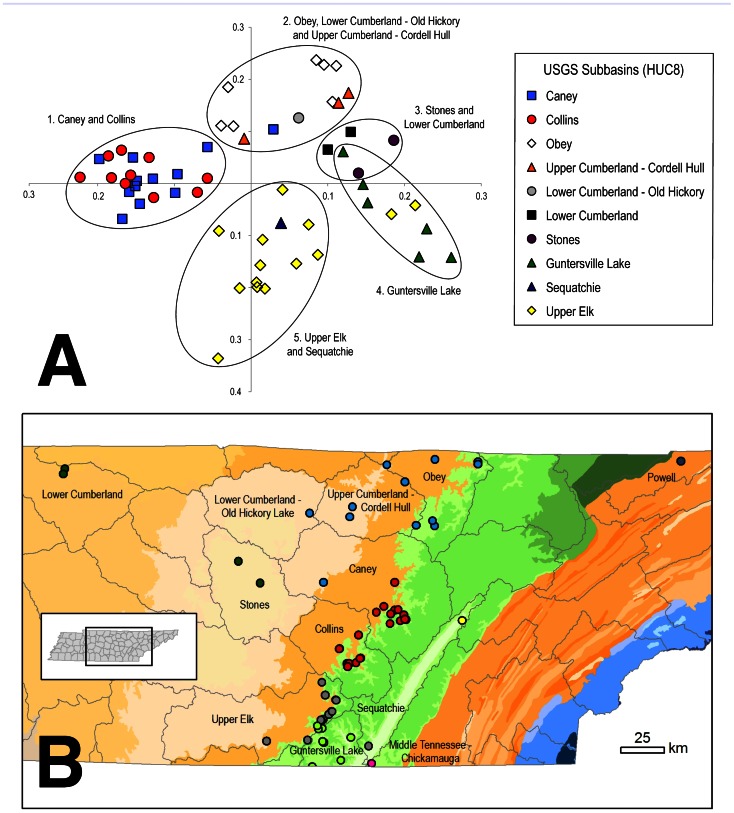
Zoogeographic regions for troglobionts of Tennessee. (a) Principal Components Analysis for 58 caves with eight or more known troglobionts. PCA Axes 1 and 2 correspond to the X and Y axes, respectively. Each point on the PCA represents a single cave, and caves with similar proximity between points indicate similarity between the troglobiont communities of those caves. Each cave is colored to reflect its location in one of the U.S. Geological Survey-defined (HUC8) watershed subbasins. Five clusters representing geographically contiguous groupings of caves are circled and labeled. (b) Locations of caves color-coded by their clusters on the PCA. Also included are three caves (one each from the Powell, Sequatchie, and Middle Tennessee–Chickamauga subbasins) that were excluded from the PCA analysis as extreme outliers and that had highly distinct troglobiont communities. Watershed boundaries are overlain on ecoregions colored as in Figure 1.

PCA and DCA identified similar regional structure in troglobiont communities across central Tennessee. This structure largely corresponded to USGS HUC8 watershed subbasins (Fig. 8a (PCA), DCA not shown). We identified five troglobiont communities composed of caves from one to three adjacent subbasins. Fifty-five of 58 caves clustered with caves from their respective subbasin or adjacent subbasins, and there was almost no overlap in the PCA between the five troglobiont communities (Fig. 8a). The two caves from the Upper Elk subbasin that clustered with caves from the Tennessee River-Guntersville Lake subbasin are located on the eastern side of the Upper Elk subbasin, less than 3 km from the drainage divide with the Tennessee River-Guntersville Lake subbasin (Fig. 8b).

The most extreme outlier in this analysis was TCB9 in Claiborne County in the Powell River subbasin, the only cave in the Ridge and Valley ecoregion with eight or more known troglobionts (Fig. 8b). Of the nine troglobionts known from TCB9, seven were not shared with any other cave in the analysis. The two shared species were the spider *Phanetta subterranea*, which is widely distributed across the state, and the amphipod *Crangonyx antennatus*, which is also found in two caves on the eastern escarpment of the Cumberland Plateau: TMN26 in the Middle Tennessee River–Chickamauga subbasin and TBD1 at the northeast corner of the Sequatchie River subbasin (Fig. 8b). These two caves were also the next greatest outliers in the analysis.

## Discussion

### Spatial Patterns of Species Richness and Endemism within Tennessee

Tennessee possesses a remarkable diversity of cave-obligate organisms, matched by only Texas with respect to overall species richness, while having more terrestrial troglobionts than any other state in the United States [1]. Two major karst regions, the Appalachians and the Interior Low Plateau, occur in Tennessee [62], [63]. These karst regions extend across multiple states and contain more caves and troglobionts than any other cave regions in the country [1], [63]. The Appalachians cave region in Tennessee is represented by the Ridge and Valley ecoregion, whereas the Interior Low Plateau cave region is represented by two ecoregions, the Interior Plateau and Southwestern Appalachians. Each of these ecoregions supports a significant troglobiont community. Although the list of known cave obligate species in Tennessee is not complete, several significant patterns have emerged from our study.

First, species richness is not evenly distributed among the major cave regions in the state (e.g., the IP, SWA and RV). Instead, it is clustered with highest richness in the southern section of the Cumberland Plateau (in the IP and SWA), with a maximum of 36 terrestrial troglobionts and eight stygobionts in a single 20×20 km grid cell. Although species richness was equivalent between the IP and SWA, species richness in the RV was less than half that observed in the other two ecoregions. This disparity is somewhat surprising given that species richness is comparable between the Interior Low Plateau, which includes the IP and SVA, and the Appalachians cave region, which includes the RV [1], [62], [63]. This is best explained by decreased availability of cave and karst habitat in the RV compared to IP and SWA in Tennessee, given that species richness is strongly associated with the number of caves observed (a proxy for available habitat; [7], this study). There are 1469 documented caves in the RV versus 5011 and 2834 caves in the IP and SWA, respectively (Table 1). Cave density (and species richness) dramatically declines in the southern RV of Tennessee where thickness and extent of exposed carbonate rocks are reduced (Figs. 1 and 3).

Only a small fraction (7.5%, 15 species) of troglobionts in the state is shared between the IP, SWA, and RV. Of the troglobionts that occur in all three ecoregions, most have broad distributions comprising multiple states, such as the pseudoscorpion *Hesperochernes mirabilis* and the cave spider *Phanetta subterranea* [64]. Beyond these fifteen species, the IP and SWA shared another 44 species, whereas the RV shared just three additional species with either the IP or SWA. Overall, the IP and SWA share many troglobionts, whereas the RV fauna is largely distinct from both.

In addition to the major differences in troglobiont diversity between ecoregions, we identified hydrological basins as another important influence on cave biodiversity in Tennessee. Troglobiont communities more closely reflect hydrological boundaries than ecoregion boundaries; indeed, most troglobiont communities included caves from the SWA and the adjacent IP (Fig. 8b). This overlap helps explain the large number of species shared between the SWA and IP. This pattern is consistent with a biogeographic break in cave communities previously observed between hydrological basins in south-central Tennessee [27]. Additional sampling is required to determine how troglobiont communities vary across the RV.

Most subterranean diversity in Tennessee caves is found regionally rather than locally within individual caves. Most caves contain but a small fraction of the regional diversity within a 20×20 km grid cell. This low alpha- versus high beta-diversity appears to be the rule rather than the exception in subterranean assemblages [2], [65]–[67]. Low levels of connectivity among caves and reduced opportunities for or abilities to disperse may result in substantially lower local diversity than regional diversity [65].

Another spatial pattern that emerged is that there is a general decline in species richness from south to north in the eastern IP and SWA, particularly along the western escarpment of the Cumberland Plateau (Fig. 3a), even though cave densities are higher to the north (Fig. 1b). This pattern has been documented previously in the Interior Low Plateau cave region by Culver et al. [17], who identified a midlatitude ridge between 33° and 35°N in North America where terrestrial subterranean biodiversity peaks. The primary hotspot of species richness (and endemism; see below) in Tennessee lies just to the north of this hypothesized ridge, with several groups reaching their highest diversity in this area, such as amphipods, millipedes, and collembolans. In contrast, the diversity gradient declines north to south in the RV, but corresponds with the gradient in cave density in this ecoregion.

Endemism, like species richness, is not homogenous nor is it concentrated in peripheral or isolated cave regions. Rather endemism is higher in Tennessee within areas that also have higher species richness. In particular, endemism was concentrated in the southern section of the Cumberland Plateau, where 25% (12 species) of single-site endemics are found in just six 20×20 km grid cells in Franklin, Grundy, and Marion counties (Fig. 5). This region is contiguous with an area of high endemism identified previously in adjacent northeastern Alabama [68]. Interestingly, endemism is not highest in regions with the greatest potential for isolation. The RV ecoregion is more dissected and less contiguous (i.e., greater potential for isolation) than the subregions of the IP and Plateau Escarpment of the SWA, yet the number of single-cave endemic species in the RV is considerably lower (Table 4).

Most cave-obligate species are known from just a few localities and few species have been reported across larger areas giving the impression that endemism is high. While levels of endemism may be overestimated due to incomplete sampling or invalid taxonomy, high endemism in subterranean fauna is a common pattern. Most terrestrial and aquatic species have small geographic ranges, with just a small fraction having large distributions [64]. Of those species with presumably large distributions, molecular studies have shown that several are actually comprised of morphologically cryptic lineages with significantly smaller ranges [69]–[71], including the lone cavefish species found in Tennessee [40]. *Typhlichthys subterraneus* is the fourth most abundant cave-obligate species in Tennessee in terms of number of localities. However, recent molecular work indicates that this species is actually comprised of several cryptic lineages (seven lineages present in Tennessee) with smaller geographic ranges that are largely isolated because of hydrological barriers [40]. Less than 10% of Tennessee’s troglobionts have been subjects of molecular or phylogeographic studies. Consequently, there is a high likelihood that additional cryptic biodiversity will be discovered in the future.

### Processes Underlying Patterns of Biodiversity and Endemism

Differences in spatial patterns of biodiversity and endemism among subterranean communities suggest that they are governed by different factors, including habitat availability, opportunity for dispersal, historical factors, and surface productivity. These factors are not mutually exclusive and multiple factors likely contribute to present patterns of biodiversity and endemism. Additional distributional data and study are needed to unravel the contributions of hypothesized underlying processes that have resulted in these patterns of diversity and endemism in subterranean communities of the Interior Low Plateau and Appalachians. However, Tennessee’s remarkable subterranean diversity may be largely explained by (1) the large amount of exposed karst and cave development but also a varied topography and geology, (2) a geographic location at the junction of the two karst regions in North America with the greatest troglobiont diversity, and (3) its proximity to the proposed mid-latitude biodiversity ridge for terrestrial cave fauna, a hypothesized region of long-term high productivity and favorable climate [17]. Below, we speculate on the importance of these processes that underlie the observed spatial patterns of subterranean biodiversity in the state.

The greater the amount of available habitat, the greater likelihood of supporting higher species richness, as there is greater potential to support larger populations and for lower extinction rates [17], [64]. Larger areas of karst, like the Interior Low Plateau, are expected to support greater numbers of species due to more caves and greater habitat diversity [8]. Species richness is highest along the western escarpment of the Cumberland Plateau in the Interior Low Plateau, which coincides with the region of greatest cave density. Previous studies have also shown that the number of caves is a good predictor of regional species richness [17], [63], [64]. In the Appalachians cave region, both cave density and species richness are lower. Increased cave density may also provide more opportunities for colonization of subterranean habitats [17].

Differences in cave connectivity and opportunities for dispersal also likely influence patterns of subterranean biodiversity. Assuming cave density is positively correlated with cave connectivity, areas of high cave density presumably have higher connectivity between caves, which offer greater opportunities for dispersal. Dispersal may decrease extinction rates and differences in species composition among localities or regions [72]. Our identification of five troglobiont communities in the SWA and IP of central Tennessee is consistent with this hypothesis, as geographically proximate caves had more similar troglobiont communities. These communities corresponded to hydrological boundaries and likely reflect increased past or current connectivity between subterranean habitats within and barriers between drainages, given most caves in the IP and SWA are solutional caves formed from dissolution of limestones and dolomites by carbonic acid dissolved in rainwater and groundwater. The greater connectivity and dispersal between the IP and SWA may explain the large number of shared species between these regions. In contrast, less connectivity may promote differences in species composition and endemism. The faulted and folded cave-bearing rock layers of the RV are more dissected and much less contiguous than the horizontal strata of the Interior Low Plateau (IP and SWA), which offer greater probability of isolation. While few species are shared between the Appalachians and Interior Low Plateau cave regions in Tennessee, endemism is actually lower in the Appalachians ([68], this study). Variation in cave connectivity and opportunities for dispersal is a plausible hypothesis to explain differences in species composition but it cannot alone explain differences in endemism among regions. However, most troglobionts in Tennessee are known from a small number of caves and have small geographic distributions, so unquestionably cave connectivity and dispersal play significant roles in shaping spatial patterns of diversity and endemism in subterranean faunas.

Differences in regional species diversity and endemism are also likely influenced by past and current environmental factors, such as climatic shifts during the Pleistocene and variation in surface productivity among regions. In North America, the cave region (southern section of the Interior Low Plateau) with greatest biodiversity is associated with high precipitation and temperature relative to most other cave regions [17]. Because almost all available food in cave systems results from surface productivity, it has been suggested that this hotspot of terrestrial cave biodiversity, which the southern Tennessee border lies just to the north of, could correspond to long-term levels of high surface productivity, particularly over recent geological times in the Pleistocene [17]. On average, caves in this region likely have more energy available to support larger populations, more species, and more diverse communities. Indeed, almost all caves with the most taxonomically diverse communities occur in this region. Southern sections of the Interior Low Plateau in Tennessee may not have experienced significant decreases in surface productivity compared to areas further north and in the Appalachians cave region, which experienced cooler temperatures and faced more dramatic dry episodes during the Pleistocene. Such rapid climatic shifts likely caused many species, particularly terrestrial species, to be extirpated or to go extinct in these regions.

### Hotspots of Subterranean Biodiversity

As originally defined [73], [74], hotspots of biodiversity are large regions of significant species richness and endemism that are also under threat at a global scale. However, hotspots are also delineated at regional and local scales to assist in setting conservation priorities. Previous studies of subterranean biodiversity have identified regional hotspots based on species richness, endemism or rarity [7], [68], [75]–[78]. We identified a hotspot of subterranean biodiversity with a center along the escarpment of the Cumberland Plateau in northeastern Franklin, southwestern Grundy, and western Marion counties defined by both species richness and endemism. Centers of both terrestrial and aquatic troglobiont diversity and endemism occur within this hotspot. This hotspot extends northward along the western escarpment of the plateau into Van Buren County and is contiguous to the south with a hotspot previously identified from Jackson County, Alabama [8], [17]. This hotspot, comprising less than 5% of Tennessee’s total area, hosts nearly 50% (91 of 200 species) of Tennessee’s subterranean biodiversity, including 71 terrestrial troglobionts and 20 stygobionts.

Although subterranean diversity is greater at regional versus local scales, conservation efforts for subterranean fauna often start with the protection of individual caves. Culver and Sket [65] were the first to identify hotspots of subterranean biodiversity at the level of individual caves. They documented 18 caves and two karst wells that contain 20 or more species of troglobionts, but this list has since increased to 36 sites [2]. Of these, just six sites occur in North America, including Shelta Cave in Madison Co., Alabama (24 species), and the Mammoth Cave system in Edmonson Co., Kentucky (41 species). Here, we add Crystal Cave in Grundy County to Culver and Pipan’s [2] list of biologically diverse caves. Crystal Cave supports 23 troglobionts, more than any other cave in the state. However, several additional caves in Franklin, Grundy, and Marion counties may reach or surpass 20 species with additional sampling effort (Table 5).

### Knowledge Gaps and Implications for Conservation and Management

The identification and protection of priority areas are common goals in managing and conserving biodiversity. However, our knowledge of subterranean biodiversity is inconsistent and often deficient in many areas. With few exceptions, cave ecosystems and habitats are poorly sampled when compared with surface ecosystems. For instance, less than 7% of caves in Tennessee have been sampled. The majority of species are known from just a few occurrences (Fig. 6). However, sampling in caves and other subterranean habitats is difficult and often directed at specific taxa (e.g., for molecular studies). Consequently, spatial coverage and sampling effort are undeniably variable among groups and it is extremely difficult to determine whether a given species is actually rare or presumed rarity is the consequence of inadequate sampling. Regardless, we identified several grid cells that are undersampled relative to the rest of the state, particularly in the northern RV of northeast Tennessee (Fig. 7).

Moreover, cave biological inventories are often plagued by uncertain taxonomy or species determination. We excluded 147 occurrence records for these very reasons, including records to 60 species reported as new or undescribed and awaiting description in the literature. This list includes up to 19 potentially new species of beetles, 11 collembolans, eight diplurans, and seven amphipods. Species rarefaction curves of expected species richness also suggest that substantial diversity remains to be sampled from each major ecoregion (Fig. 4; Table 4), particularly for terrestrial species. The evidence strongly suggests that many additional species await discovery from subterranean habitats in Tennessee.

To this end, we outline six conservation and management priorities related to subterranean fauna in the Interior Low Plateau and Appalachians cave regions of Tennessee: (1) Improve the spatial coverage by sampling caves in areas identified as undersampled, in particular the Ridge and Valley ecoregion of northeast Tennessee. (2) Improve the sampling effort for diverse taxonomic groups, particularly *Pseudanophthalmus* beetles, *Litocampa* diplurans, and *Stygobromus* amphipods, where most species are known from just a few caves and numerous undescribed species have been reported. (3) Conduct molecular work on widespread species (e.g., the isopod *Caecidotea bicrenata*, the spider *Phanetta subterranea* and the fly *Spelobia tenebrarum*) to determine whether these taxa contain cryptic lineages and diversity. (4) Work with taxonomic specialists to describe the 60 taxa reported as new or undescribed in the literature. (5) Increase the geographic extent of the database to include cave regions in adjacent states in order to improve our knowledge on geographic distributions of individual species as well as estimates of species richness and endemism at varying spatial scales. (6) Conduct conservation assessments when data warrant on troglobionts of Tennessee. Just 10 of the 200 species reported in Tennessee have had International Union for the Conservation of Nature (IUCN) Red List assessments conducted (Table S1). Despite recent progress, significant work remains to clarify the ecology and evolution of Tennessee’s cave ecosystems.

## Supporting Information

Figure S1 **Cumulative number of new species of troglobionts reported from Tennessee since 1840.** The dashed line shows the number of species described by decade.
(TIF)

Figure S2 **Spatial patterns of species richness and endemism in Tennessee counties.** (a) Counties of Tennessee, (b) troglobionts per county, and (c) single-site endemics per county.
(TIF)

Figure S3 **Species accumulation curves for the major cave-bearing Level IV ecoregions (subregions of Level III ecoregions in Tennessee, including (a) Western Highland Rim, (b) Western Pennyroyal Karst, (c) Outer Nashville Basin, (d) Inner Nashville Basin, (e) Eastern Highland Rim, and (f) Plateau Escarpment.** Species accumulation curves are shown for all troglobionts (gray), terrestrial troglobionts (red), and stygobionts (blue). The shaded area around each line represents the 95% confidence interval.
(TIF)

Table S1 List of described troglobionts, including 160 terrestrial troglobionts and 40 stygobionts, documented from Tennessee caves and associated habitats.
(DOCX)

Dataset S1 **CSV data file of presence-absence matrix of caves and species used in statistical analyses.** The working dataset included 1976 records representing 661 caves and 196 troglobionts.
(CSV)

Text S1 **Bibliography of Tennessee cave obligate species.**
(DOC)
