# Supplementary figures and images for: Patterns of Cave Biodiversity and Endemism in the Appalachians and Interior Plateau of Tennessee, USA
Source: PLoS One. 2013 May 22;8(5):e64177. doi: 10.1371/journal.pone.0064177 (PMC3661478; doi:10.1371/journal.pone.0064177)

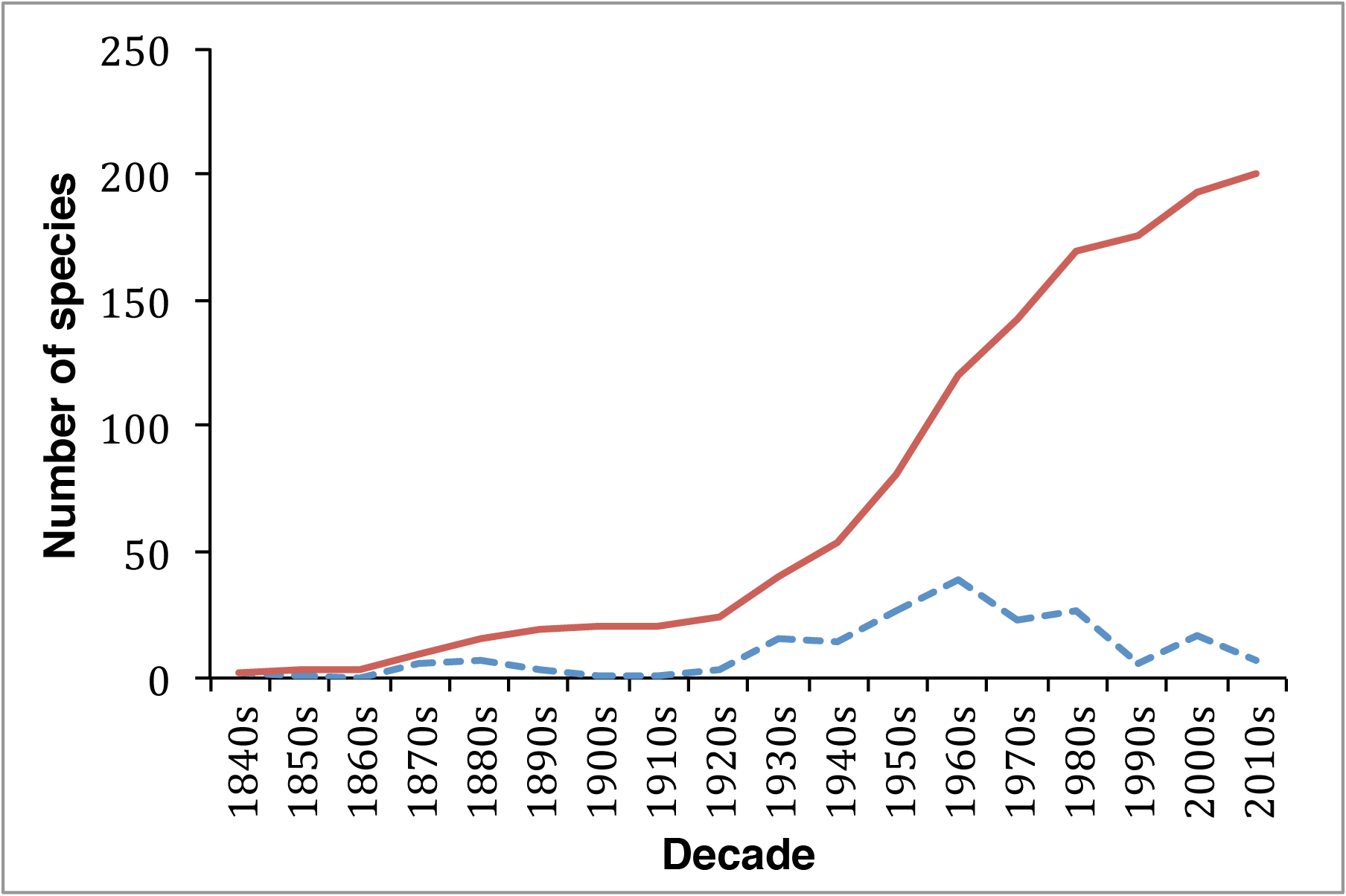

Supplement: Figure S1 — Cumulative number of new species of troglobionts reported from Tennessee since 1840. The dashed line shows the number of species described by decade. (TIF) [file pone.0064177.s001.tif]

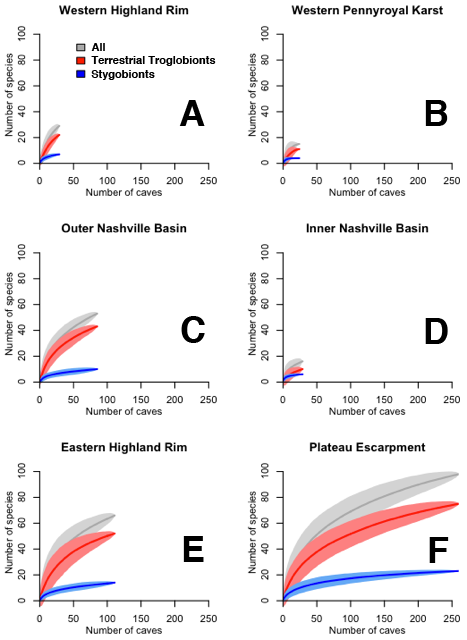

Supplement: Figure S3 — Species accumulation curves for the major cave-bearing Level IV ecoregions (subregions of Level III ecoregions in Tennessee, including (a) Western Highland Rim, (b) Western Pennyroyal Karst, (c) Outer Nashville Basin, (d) Inner Nashville Basin, (e) Eastern Highland Rim, and (f) Plateau Escarpment. Species accumulation curves are shown for all troglobionts (gray), terrestrial troglobionts (red), and stygobionts (blue). The shaded area around each line represents the 95% confidence interval. (TIF) [file pone.0064177.s003.tif]
