# Supplementary material for: Patterns of Cave Biodiversity and Endemism in the Appalachians and Interior Plateau of Tennessee, USA
Source: PLoS One. 2013 May 22;8(5):e64177. doi: 10.1371/journal.pone.0064177 (PMC3661478; doi:10.1371/journal.pone.0064177)
Supplement: Table S1 — List of described troglobionts, including 160 terrestrial troglobionts and 40 stygobionts, documented from Tennessee caves and associated habitats. (DOCX) [file pone.0064177.s004.docx]

**Table S1.** List of described troglobionts, including 160 troglobites and 40 stygobites, documented from Tennessee caves and associated habitats.

| Species | Authority | Ecological Classification | IUCN Red List |
| --- | --- | --- | --- |
| **Phylum Annelida** |  |  |  |
| **Class Clitellata** |  |  |  |
| **Order Branchiobdellida** |  |  |  |
| **Family Cambarincolidae** |  |  |  |
| *Cambarincola alienus* | Holt, 1963 | SB |  |
| *Cambarincola marthae* | Holt, 1973 | SB |  |
| **Order Lumbriculida** |  |  |  |
| **Family Lumbriculidae** |  |  |  |
| *Eremidrilus allegheniensis* | (Cook, 1971) | SB |  |
| **Phylum Platyhelminthes** |  |  |  |
| **Class Turbellaria** |  |  |  |
| **Order Tricladida** |  |  |  |
| **Family Kenkiidae** |  |  |  |
| *Sphalloplana chandleri* | Kenk, 1977 | SB |  |
| *Sphalloplana consimilis* | Kenk, 1977 | SB |  |
| *Sphalloplana percoeca* | (Packard, 1879) | SB |  |
| **Phylum Mollusca** |  |  |  |
| **Class Gastropoda** |  |  |  |
| **Order Basommatophora** |  |  |  |
| **Family Carychiidae** |  |  |  |
| *Carychium stygium* | Call, 1897 | TB |  |
| **Order Stylommatophora** |  |  |  |
| **Family Helicodiscidae** |  |  |  |
| *Helicodiscus barri* | Hubricht, 1962 | TB |  |
| *Helicodiscus notius* | Hubricht, 1962 | TB |  |
| **Family Zonitidae** |  |  |  |
| *Glyphyalinia specus* | Hubricht, 1965 | TB |  |
| **Phylum Arthropoda** |  |  |  |
| **Class Arachnida** |  |  |  |
| **Order Acari** |  |  |  |
| **Family Rhagidiidae** |  |  |  |
| *Poecilophysis weyerensis* | (Packard, 1888) | TB |  |
| **Order Araneae** |  |  |  |
| **Family Linyphiidae** |  |  |  |
| *Anthrobia monouthia* | Tellkampf, 1844 | TB |  |
| *Oreonetides beattyi* | Paquin et al., 2009 | TB |  |
| *Phanetta subterranea* | (Emerton, 1875) | TB |  |
| *Porrhomma cavernicola* | (Keyserling, 1886) | TB |  |
| **Family Nesticidae** |  |  |  |
| *Nesticus barri* | Gertsch, 1984 | TB |  |
| *Nesticus barrowsi* | Gertsch, 1984 | TB |  |
| *Nesticus dilutus* | Gertsch, 1984 | TB |  |
| *Nesticus furtivus* | Gertsch, 1984 | TB |  |
| *Nesticus paynei* | Gertsch, 1984 | TB |  |
| *Nesticus pecki* | Hedin and Dellinger, 2005 | TB |  |
| *Nesticus stygius* | Gertsch, 1984 | TB |  |
| *Nesticus tennesseensis* | (Petrunkevich, 1925) | TB |  |
| **Family Tengellidae** |  |  |  |
| *Liocranoides archeri* | Platnick, 1999 | TB |  |
| *Liocranoides unicolor* | Keyserling, 1886 | TB |  |
| **Order Opiliones** |  |  |  |
| **Family Phalangodidae** |  |  |  |
| *Tolus appalachius* | (Goodnight and Goodnight, 1942) | TB |  |
| **Order Pseudoscorpiones** |  |  |  |
| **Family Chernetidae** |  |  |  |
| *Hesperochernes mirabilis* | (Banks, 1895) | TB |  |
| **Family Chthoniidae** |  |  |  |
| *Apochthonius minor* | Muchmore, 1976 | TB |  |
| *Kleptochthonius affinis* | Muchmore, 1976 | TB |  |
| *Kleptochthonius barri* | Muchmore, 1965 | TB |  |
| *Kleptochthonius charon* | Muchmore, 1965 | TB |  |
| *Kleptochthonius daemonius* | Muchmore, 1965 | TB |  |
| *Kleptochthonius infernalis* | Malcolm and Chamberlin, 1961 | TB |  |
| *Kleptochthonius magnus* | Muchmore, 1966 | TB |  |
| *Kleptochthonius myopius* | Malcolm and Chamberlin, 1961 | TB |  |
| *Kleptochthonius pluto* | Muchmore, 1965 | TB |  |
| *Kleptochthonius rex* | Malcolm and Chamberlin, 1961 | TB |  |
| *Kleptochthonius stygius* | Muchmore, 1965 | TB |  |
| *Kleptochthonius tantalus* | Muchmore, 1966 | TB |  |
| *Tyrannochthonius fiskei* | Muchmore, 1996 | TB |  |
| *Tyrannochthonius halopotamus* | Muchmore, 1996 | TB |  |
| *Tyrannochthonius steevesi* | Muchmore, 1996 | TB |  |
| **Family Neobisiidae** |  |  |  |
| *Lissocreagris nickajackensis* | (Muchmore, 1966) | TB |  |
| **Family Syarinidae** |  |  |  |
| *Chitrella archeri* | Malcolm and Chamberlin, 1960 | TB |  |
| **Class Diplopoda** |  |  |  |
| **Order Callipodida** |  |  |  |
| **Family Abacionidae** |  |  |  |
| *Tetracion jonesi* | Hoffman, 1956 | TB |  |
| *Tetracion tennesseensis* | Causey, 1959 | TB |  |
| **Order Chordeumatida** |  |  |  |
| **Family Cleidogonidae** |  |  |  |
| *Pseudotremia acheron* | Shear, 1972 | TB |  |
| *Pseudotremia barri* | Lewis 2005 | TB |  |
| *Pseudotremia callae* | Lewis 2009 | TB |  |
| *Pseudotremia cercops* | Shear, 1972 | TB |  |
| *Pseudotremia douglasi* | Lewis 2009 | TB |  |
| *Pseudotremia eburnea* | Loomis, 1939 | TB |  |
| *Pseudotremia garlandae* | Lewis 2005 | TB |  |
| *Pseudotremia hoffmani* | Lewis 2009 | TB |  |
| *Pseudotremia hollidayi* | Lewis 2005 | TB |  |
| *Pseudotremia lethe* | Shear, 1972 | TB |  |
| *Pseudotremia lictor* | Shear, 1972 | TB |  |
| *Pseudotremia manni* | Lewis 2005 | TB |  |
| *Pseudotremia minos* | Shear, 1972 | TB |  |
| *Pseudotremia nodusa* | Loomis, 1939 | TB |  |
| *Pseudotremia rhadamanthus* | Shear, 1972 | TB |  |
| *Pseudotremia roebuckorum* | Lewis 2005 | TB |  |
| *Pseudotremia sheari* | Lewis 2009 | TB |  |
| *Pseudotremia valga* | Loomis, 1943 | TB |  |
| *Pseudotremia wallaceae* | Lewis 2005 | TB |  |
| *Pseudotremia withersi* | Lewis 2009 | TB |  |
| *Scoterpes blountensis* | Shear, 2010 | TB |  |
| *Scoterpes copei* | (Packard, 1881) | TB |  |
| *Scoterpes hesperus* | Shear, 2010 | TB |  |
| *Scoterpes jackdanieli* | Shear, 2010 | TB |  |
| *Scoterpes musicarustica* | Shear, 2010 | TB |  |
| *Scoterpes stewartpecki* | Shear, 2010 | TB |  |
| *Scoterpes tombarri* | Shear, 2010 | TB |  |
| *Scoterpes tricorner* | Shear, 2010 | TB |  |
| *Scoterpes ventus* | Shear, 1972 | TB |  |
| **Order Julida** |  |  |  |
| **Family Zosteractinidae** |  |  |  |
| *Ameractis satis* | Causey, 1959 | TB |  |
| **Order Polydesmida** |  |  |  |
| **Family Macrosternodesmidae** |  |  |  |
| *Chaetaspis debilis* | (Causey, 1959) | TB |  |
| *Chaetaspis mollis* | (Causey, 1959) | TB |  |
| **Class Hexapoda** |  |  |  |
| **Order Collembola** |  |  |  |
| **Family Entomobryidae** |  |  |  |
| *Pseudosinella christianseni* | Salmon, 1964 | TB |  |
| *Pseudosinella hirsuta* | (Delamare Deboutteville, 1949) | TB |  |
| *Pseudosinella orba* | Christiansen, 1961 | TB |  |
| *Pseudosinella pecki* | Christiansen and Bonet, 1980 | TB |  |
| *Pseudosinella spinosa* | (Delamare Deboutteville, 1949) | TB |  |
| *Sinella basidens* | Bonet, 1934 | TB |  |
| *Sinella cavernarum* | (Packard, 1881) | TB |  |
| **Family Sminthuridae** |  |  |  |
| *Arrhopalites marshalli* | Christiansen and Bellinger, 1996 | TB |  |
| *Arrhopalites pavo* | Christiansen and Bellinger, 1996 | TB |  |
| **Family Tomoceridae** |  |  |  |
| *Tomocerus missus* | Mills, 1948 | TB |  |
| **Order Diplura** |  |  |  |
| **Family Campodeidae** |  |  |  |
| *Litocampa cookei* | (Packard, 1871) | TB |  |
| *Litocampa jonesi* | Conde, 1949 | TB |  |
| *Litocampa valentinei* | (Conde, 1949) | TB |  |
| **Class Insecta** |  |  |  |
| **Order Coleoptera** |  |  |  |
| **Family Carabidae** |  |  |  |
| *Anillinus longiceps* | Jeannel, 1963 | TB |  |
| *Darlingtonea kentuckensis* | Valentine, 1952 | TB |  |
| *Nelsonites walteri* | Valentine, 1952 | TB |  |
| *Pseudanophthalmus acherontis* | Barr, 1959 | TB |  |
| *Pseudanophthalmus beakleyi* | Valentine, 1937 | TB |  |
| *Pseudanophthalmus bendermani* | Barr, 1959 | TB |  |
| *Pseudanophthalmus catherinae* | Barr, 1959 | TB |  |
| *Pseudanophthalmus cerberus cerberus* | Barr, 1985 | TB |  |
| *Pseudanophthalmus ciliaris* | Valentine, 1937 | TB |  |
| *Pseudanophthalmus colemanensis* | Barr, 1959 | TB |  |
| *Pseudanophthalmus cumberlandus* | Valentine, 1937 | TB |  |
| *Pseudanophthalmus digitus* | Valentine, 1932 | TB |  |
| *Pseudanophthalmus englehardti* | (Barber, 1928) | TB |  |
| *Pseudanophthalmus farrelli* | (Barr, 1959) | TB |  |
| *Pseudanophthalmus fowlerae* | Barr, 1980 | TB |  |
| *Pseudanophthalmus fulleri* | Valentine, 1932 | TB |  |
| *Pseudanophthalmus hesperus* | Barr, 1959 | TB |  |
| *Pseudanophthalmus hirsutus* | Valentine, 1931 | TB |  |
| *Pseudanophthalmus humeralis* | Valentine, 1931 | TB |  |
| *Pseudanophthalmus inquisitor* | Barr, 1980 | TB |  |
| *Pseudanophthalmus insularis* | Barr, 1959 | TB |  |
| *Pseudanophthalmus intermedius* | (Valentine, 1931) | TB |  |
| *Pseudanophthalmus jonesi* | Valentine, 1945 | TB |  |
| *Pseudanophthalmus loganensis* | Barr, 1959 | TB |  |
| *Pseudanophthalmus longiceps* | Barr, 1951 | TB |  |
| *Pseudanophthalmus macradyi* | Valentine, 1948 | TB |  |
| *Pseudanophthalmus nickajackensis* | Barr, 1981 | TB |  |
| *Pseudanophthalmus nortoni* | Barr, 1981 | TB |  |
| *Pseudanophthalmus occidentalis* | Barr, 1959 | TB |  |
| *Pseudanophthalmus pallidus* | Barr, 1981 | TB |  |
| *Pseudanophthalmus paradoxus* | Barr, 1981 | TB |  |
| *Pseudanophthalmus paulus* | Barr, 1981 | TB |  |
| *Pseudanophthalmus paynei* | Barr, 1981 | TB |  |
| *Pseudanophthalmus princeps* | Barr, 1979 | TB |  |
| *Pseudanophthalmus productus* | Barr, 1980 | TB |  |
| *Pseudanophthalmus pusillus* | Barr, 1981 | TB |  |
| *Pseudanophthalmus robustus* | Valentine, 1931 | TB |  |
| *Pseudanophthalmus rotundatus* | Valentine, 1932 | TB |  |
| *Pseudanophthalmus scutilis* | Barr, 1981 | TB |  |
| *Pseudanophthalmus sidus* | Barr, 1965 | TB |  |
| *Pseudanophthalmus simplex* | Barr, 1980 | TB |  |
| *Pseudanophthalmus templetoni* | Valentine, 1948 | TB |  |
| *Pseudanophthalmus tennesseensis* | Valentine, 1937 | TB |  |
| *Pseudanophthalmus tiresias* | Barr, 1959 | TB |  |
| *Pseudanophthalmus tullahoma* | Barr, 1959 | TB |  |
| *Pseudanophthalmus unionis* | Barr, 1981 | TB |  |
| *Pseudanophthalmus valentinei* | Jeannel, 1949 | TB |  |
| *Pseudanophthalmus vanburenensis* | Barr, 1959 | TB |  |
| *Pseudanophthalmus ventus* | Barr, 1981 | TB |  |
| *Pseudanophthalmus wallacei* | Barr, 1981 | TB |  |
| **Family Leiodidae** |  |  |  |
| *Ptomaphagus barri* | Peck, 1973 | TB |  |
| *Ptomaphagus chromolithus* | Peck, 1984 | TB |  |
| *Ptomaphagus fecundus* | Barr, 1963 | TB |  |
| *Ptomaphagus hatchi* | Jeannel, 1933 | TB |  |
| *Ptomaphagus hubrichti* | Barr, 1959 | TB |  |
| **Family Staphylinidae** |  |  |  |
| *Arianops gorei* | Carlton, 2008 | TB |  |
| *Arianops pecki* | Barr, 1974 | TB |  |
| *Arianops stygica* | Park, 1960 | TB |  |
| *Batriasymmodes greeveri* | Park, 1965 | TB |  |
| *Batriasymmodes quisnamus* | (Park, 1951) | TB |  |
| *Batriasymmodes spelaeus* | (Park, 1951) | TB |  |
| *Batrisodes barri* | Park, 1958 | TB |  |
| *Batrisodes clypeospecus* | Park, 1951 | TB |  |
| *Batrisodes ferulifer* | Park, 1951 | TB |  |
| *Batrisodes gemmoides* | Park, 1960 | TB |  |
| *Batrisodes gemmus* | Park, 1956 | TB |  |
| *Batrisodes pannosus* | Park, 1960 | TB |  |
| *Batrisodes specus* | Park, 1951 | TB |  |
| *Batrisodes valentinei* | Park, 1951 | TB |  |
| *Subterrochus ferus* | (Park, 1951) | TB |  |
| *Subterrochus steevesi* | (Park, 1960) | TB |  |
| *Tychobythinus strinatii* | Besuchet, 1982 | TB |  |
| **Order Diptera** |  |  |  |
| **Family Sphaeroceridae** |  |  |  |
| *Spelobia tenebrarum* | (Aldrich, 1897) | TB |  |
| **Class Malacostraca** |  |  |  |
| **Order Amphipoda** |  |  |  |
| **Family Crangonyctidae** |  |  |  |
| *Bactrurus angulus* | Koenemanna and Holsinger, 2001 | SB |  |
| *Crangonyx antennatus* | Cope and Packard, 1881 | SB |  |
| *Stygobromus alabamensis* | (Stout, 1911) | SB |  |
| *Stygobromus barryi* | Holsinger, 1978 | SB |  |
| *Stygobromus dicksoni* | Holsinger, 1978 | SB |  |
| *Stygobromus exilis* | Hubricht, 1943 | SB |  |
| *Stygobromus fecundus* | Holsinger, 1978 | SB |  |
| *Stygobromus finleyi* | Holsinger, 1978 | SB |  |
| *Stygobromus mackini* | Hubricht, 1943 | SB |  |
| *Stygobromus nortoni* | (Holsinger, 1969) | SB | VU D2 |
| *Stygobromus sparsus* | Holsinger, 1978 | SB |  |
| *Stygobromus vitreus* | Cope, 1872 | SB |  |
| **Order Decapoda** |  |  |  |
| **Family Cambaridae** |  |  |  |
| *Cambarus hamulatus* | (Cope, 1881) | SB | LC |
| *Orconectes australis* | (Rhoades, 1941) | SB | LC |
| *Orconectes barri* | Buhay and Crandall, 2008 | SB | DD |
| *Orconectes incomptus* | Hobbs and Barr, 1972 | SB | VU D2 |
| *Orconectes pellucidus* | (Tellkampf, 1844) | SB | LC |
| **Order Isopoda** |  |  |  |
| **Family Asellidae** |  |  |  |
| *Caecidotea bicrenata* | (Steeves, 1963) | SB |  |
| *Caecidotea circulus* | (Steeves, 1968) | SB |  |
| *Caecidotea incurva* | (Steeves, 1968) | SB |  |
| *Caecidotea nickajackensis* | Packard, 1881 | SB | VU D2 |
| *Caecidotea nortoni* | (Steeves, 1966) | SB |  |
| *Caecidotea recurvata* | (Steeves, 1963) | SB |  |
| *Caecidotea richardsonae* | Hay, 1901 | SB |  |
| *Caecidotea scyphus* | (Steeves and Holsinger, 1968) | SB |  |
| *Caecidotea stygia* | Packard, 1871 | SB |  |
| **Family Ligiidae** |  |  |  |
| *Ligidium elrodii hancockensis* | Schultz, 1970 | TB |  |
| **Family Trichoniscidae** |  |  |  |
| *Amerigoniscus nicholasi* | (Vandel, 1965) | TB |  |
| **Class Maxillopoda** |  |  |  |
| **Order Cyclopoida** |  |  |  |
| **Family Cyclopidae** |  |  |  |
| *Diacyclops indianensis* | Reid, 2004 | SB |  |
| *Diacyclops yeatmani* | Reid, 1988 | SB |  |
| *Megacyclops donnaldsoni* | (Chappuis, 1929) | SB |  |
| **Class Ostracoda** |  |  |  |
| **Order Podocopida** |  |  |  |
| **Family Candonidae** |  |  |  |
| *Pseudocandona jeanneli* | (Klie, 1931) | SB |  |
| **Family Entocytheridae** |  |  |  |
| *Sagittocythere barri* | (Hart and Hobbs, 1961) | SB |  |
| **Phylum Chordata** |  |  |  |
| **Class Actinopterygii** |  |  |  |
| **Order Percopsiformes** |  |  |  |
| **Family Amblyopsidae** |  |  |  |
| *Typhlichthys subterraneus* | Girard, 1859 | SB | VU D2 |
| **Class Amphibia** |  |  |  |
| **Order Caudata** |  |  |  |
| **Family Plethodontidae** |  |  |  |
| *Gyrinophilus gulolineatus* | Brandon, 1965 | SB | EN B1ab(iii)+2ab(iii) |
| *Gyrinophilus palleucus* | Macrady, 1954 | SB | VU B2ab(iii,v) |
| TB - troglobite; SB - stygobite. |  |  |  |
