# Supplementary material for: Patterns of Cave Biodiversity and Endemism in the Appalachians and Interior Plateau of Tennessee, USA
Source: PLoS One. 2013 May 22;8(5):e64177. doi: 10.1371/journal.pone.0064177 (PMC3661478; doi:10.1371/journal.pone.0064177)
Supplement: Text S1 — Bibliography of Tennessee cave obligate species. (DOC) [file pone.0064177.s006.doc]

**TN Troglobiont Database References**

Aldrich JM (1897) A collection of Diptera from Indiana caves, pp. 186-190. In: Blatchley WS, The Fauna of Indiana Caves. Ind Dept Geol and Nat Resources, Ann Rpt (1896) 21: 175–212.

Armstrong JG, Williams JD (1971) Cave and spring fishes of the southern bend of the Tennessee River. J Tenn Acad Sci 46: 107–115.

Ashton RE Jr (1986) Tennessee cave salamander. Pp. 60–62. In Mount RH (ed.). Vertebrate Animals of Alabama in Need of Special Attention. Alabama Agricultural Experimental Station, Auburn University, Auburn, AL.

Banks N (1895) Notes on the Pseudoscorpionida. J New York Entomol Soc. 3: 1–13.

Barber HS (1928) Two new cave beetles related to *Anophthalmus pusio* Horn. Jour Washington Acad Sci 18: 195–196.

Barr TC Jr (1958) A new cave beetle of the genus *Ptomaphagus* (Catopidae) from DeKalb County, Tennessee. J Tenn Acad Sci 33: 170-171.

Barr TC Jr (1959) New cave beetles (Carabidae, Trechini) from Tennessee and Kentucky. J Tenn Acad Sci 34: 5-30.

Barr TC Jr (1961) Caves of Tennessee. Tennessee Div Geol Bull 64: 1-567.

Barr TC Jr (1962) The *robustus* Group in the genus *Pseudanophthalmus* (Coleoptera: Carabidae: Trechini). Coleopt Bull 16: 109-118.

Barr TC Jr (1963) Studies on the cavernicole *Ptomaphagus* of the United States (Coleoptera: Catopidae). Psyche 70: 50-58.

Barr TC Jr (1965) The *Pseudanophthalmus* of the Appalachian Valley (Coleoptera: Carabidae). Amer Midl Natur 73: 41-72.

Barr TC Jr (1974) The eyeless beetles of the genus *Arianops* Brendel (Coleoptera, Pselaphidae). Bulletin of the American Museum of Natural History 154: 1-52.

Barr TC Jr (1979) The taxonomy, distribution, and affinities of *Neaphaenops*, with notes on associated species of *Pseudanophthalmus* (Coleoptera: Carabidae). Amer Mus Nov 2867: 1-2.

Barr TC Jr (1980) New species groups of *Pseudanophthalmus* from the Central Basin of Tennessee (Coleoptera: Carabidae: Trechinae). Brimleyana 3: 85-96.

Barr TC Jr (1981) *Pseudanophthalmus* from Appalachian caves (Coleoptera: Carabidae): the *engelhardti* complex. Brimleyana 5: 37-94.

Barr TC Jr (1985) New trechine beetles (Coleoptera: Carabidae) from the Appalachian region. Brimleyana 11: 119-132.

Barr TC Jr (2004) A Classification and Checklist of the Genus *Pseudanophthalmus* Jeannel (Coleoptera: Carabidae: Trechinae). Virginia Museum of Natural History Special Publication 11, 52 pp.

Barr TC. Jr, Holsinger J (1985) Speciation in cave faunas. Annu Rev Ecol Sys 16: 313–337.

Beachy CK (2005a) *Gyrinophilus gulolineatus*. Pp. 774–775, In Lannoo M (Ed.). Amphibian Declines: the Conservation Status of United States Species. University of California, Berkeley, CA.

Beachy CK (2005b) Gyrinophilus palleucus. Pp. 775–776. In Lannoo M (Ed.). Amphibian Declines: the Conservation Status of United States Species. University of California, Berkeley, CA.

Bechler DL (1976) *Typhlichthys subterraneus* Girard (Pisces: Amblyopsidae) in the Jackson Plain of Tennessee. Bull Nat Speleol Soc 38: 39–40.

Bechler DL (1980) The evolution of agonistic behavior in amblyopsid fishes. Ph.D. dissertation. Saint Louis University, Saint Louis, MO. 160pp.

Bechler DL (1981) Agonistic behavior in the Amblyopsidae, the spring, cave, and swamp fishes. Proc Int Congr Speleol 8: 68–69.

Bechler DL (1983) The evolution of agonistic behavior in amblyopsid fishes. Behav Ecol Sociobiol 12: 35–42.

Beharse JC, Brandon RA (1973) Optomotor response and eye structure of the troglobitic salamander Gyrinophilus palleucus. Am Midl Nat 89: 463–467.

Bergstrom DE (1997) The phylogeny and historical biogeography of Missouri’s *Amblyopsis rosae* (Ozark cavefish) and *Typhlichthys subterraneus* (southern cavefish). M.S. Thesis. University of Missouri, Columbia, MO. 63 pp.

Bergstrom DE, Noltie DB, Holtsford TP (1995) Ozark cavefish genetics: the phylogeny of Missouri’s Ozark cavefish (*Amblyopsis rosae*) and southern cavefish (*Typhlichthys subterraneus*). Technical report. Missouri Department of Conservation, Springfield, MO. 29 pp.

Besuchet C (1982) Contribution a l’etude des Bythinini cavernicoles nearctiques (Coleoptera: Pselaphidae). Rev Suisse Zool 89: 49-53.

Bonet MF (1934) Biospeleogica No. XL Campagne speleoligique de C. Bolivar et R. Jeannel dans l’Amerique du Nord. 10 Collemboles. Arch Zool Exptl et Gen 76: 361-377.

Brandon RA (1965) A new race of the neotenic salamander *Gyrinophilus palleucus*. Copeia 1965: 346-352.

Blair AP (1961) Metamorphosis of *Pseudotriton palleucus* with iodine. Copeia 1961: 499.

Brandon RA (1962) A systematic study of the salamander genus Gyrinophilus. Ph.D. dissertation, University of Illinois, Urbana, IL. 242 pp.

Brandon RA (1965) A new race of the neotenic salamander *Gyrinophilus palleucus*. Copeia 1965: 346–352.

Brandon RA (1966) Systematics of the salamander genus *Gyrinophilus*. Illinois Biol Monogr 35: 1–85.

Brandon RA (1967a) *Gyrinophilus palleucus* McCrady. Cat Am Amphibians Reptiles 32: 1–2.

Brandon RA (1967b) Food and an intestinal parasite of the troglobitic salamander *Gyrinophilus palleucus necturoides*. Herpetologica 23: 52–53.

Brandon RA (1971) North American troglobitic salamanders: some aspects of modification in cave habitats, with special reference to *Gyrinophilus palleucus*. Bull Nat Speleol Soc 33: 1–21.

Brandon RA, Jacobs J, Wynn A, Sever DM (1986) A naturally metamorphosed Tennessee cave salamander (*Gyrinophilus palleucus*). J Tenn Acad Sci 61: 1–2.

Buhay JE, Crandall KA (2005) Subterranean phylogeography of freshwater crayfishes shows extensive gene flow and surprisingly large population sizes. Molecular Ecology 14: 4259-4273.

Buhay JE, Crandall KA (2008) Taxonomic revision of cave crayfishes in the genus *Orconectes*, subgenus *Orconectes* (Decapoda: Cambaridae) along the Cumberland Plateau, including a description of a new species, *Orconectes barri*. Journal of Crustacean Biology 28: 57-67.

Buhay JE, Moni G, Mann N, Crandall KA (2007) Molecular taxonomy in the dark: Evolutionary history, phylogeography, and diversity of cave crayfish in the subgenus *Aviticambarus*, genus *Cambarus*. Molecular Phylogenetics and Evolution 42: 435-448.

Bunting DL (1973) The Cladocera and Copepoda of Tennessee. II. Cyclopoid copepods. J Tenn Acad Sci 48: 138–141.

Caldwell RS, Copeland JE (1992) Status and habitat of the Tennessee cave salamander. Technical. Report. Tennessee Wildlife Resources Agency, Nashville, TN. 24 pp.

Call RE (1897) Notes on the flora of Mammoth Cave, Kentucky. J Cincinnati Soc Nat Hist 19: 79-80.

Carlton CE (2008) Eight new species of *Arianops* Brendel from the Southeastern United States with an updated key and notes on additional species (Coleoptera: Staphylinidae: Pselaphinae). The Coleopterists Bulletin 62: 297-323.

Carpenter JH (1970) Systematics and ecology of cave planarians of the United States. 213 pp. Ph.D. dissertation, University of Kentucky.

Causey NB (1959) Some cavernicolous millipeds from the Cumberland Plateau. J Tenn Acad Sci 34: 229-237.

Chandler CM, Darlington JT (1984) Further field studies of freshwater planarians of Tennessee (Turbellaria: Tricladida): II. Middle Tennessee. J Freshwater Ecology 2: 561-570.

Chappuis PA (1929) Campagne spéologique de C. Bolivar et R. Jeannel dans l’Amérique du Nord (1928). 4. Crustacés Copépodes. Arch Zool Expér Gén 71: 345-360.

Christiansen KA (1960) The genus *Pseuedosinella* (Collembola, Entomobryidae) in caves of the United States. Psyche 67: 1-24.

Christiansen KA (1961) Convergence and parallelism in cave entomobryinae. Evolution 15: 288–301.

Christiansen KA, Bellinger P (1980) The Collembola of North American north of the Rio Grande. Part 3. Family Entomobryidae. Grinnell College, Grinnell, IA.

Christiansen KA, Bellinger P (1996) Cave *Arrhopalites*: new to science. J Cave Karst Stud. 58: 168-180.

Christiansen KA, Bellinger P (1998) The Collembola of North American North of the Rio Grande: a Taxonomic Analysis. Grinnell College, Grinnell, IA. 1520 pp.

Condé B (1949) Campodéidés cavernicoles de la région des Appalaches. Notés Biospéologiques 4: 125-137.

Cook DG (1971) *Trichodrilus allegheniensis* n. sp. (Oligochaeta, Lumbriculidae) from a cave in southern Tennessee. Trans Amer Micros Soc 90: 381-383.

Cooper JE (1968) The salamander Gyrinophilus palleucus in Georgia, with notes on Alabama and Tennessee populations. J Alabama Acad Sci 39: 182–185.

Cooper JE, Cooper MR (1968) Cave-associated herpetozoa II: salamanders of the genus *Gyrinophilus* in Alabama caves. Bull Nat Speleol Soc 30: 19–24.

Cope ED (1872) Descriptions of species from the Wyandotte Cave; also from Mammoth Cave. Rept. Indiana Geol Surv 4: 173-182.

Cope ED, Packard AS (1881) The fauna of Nickajack Cave. Amer Nat 15: 877–882.

Craig DL, Kirby-Smith JS, Dent JN (1956) Mitotic inhibition and chromosome breakage in salamanders of the genus *Gyrinophilus*. Trans Am Microscop Soc 75: 234–241.

Culver DC, Master LL, Christman MC, Hobbs III HH (2000) Obligate cave fauna of the 48 contiguous United States. Conserv Biol 14: 386–401.

Delamare C (1949) Collemboles cavernicoles du Tennessee et de L’Alabama. Notes Biospeleologiques 4: 117-124.

Dent JN, Craig DL (1955) Induction of metamorphosis in *Gyrinophilus palleucus*. Anat Rec 121: 429.

Dent JN, Kirby-Smith JS (1963) Metamorphic physiology and morphology of the cave salamander *Gyrinophilus palleucus*. Copeia 1963: 119–130.

Dillman CB, Bergstrom DE, Noltie DB, Holtsford TP, Mayden RL (2011) Regressive progression, progressive regression or neither? Phylogeny and evolution of the Percopsiformes (Teleostei, Paracanthopterygii). Zoologica Scripta 40: 45–60.

Dixon GB, Zigler KS (2011) Cave-obligate biodiversity on the campus of Sewanee: The University of the South, Franklin County, Tennessee. Southeast Nat 10: 251–266.

Eigenmann CH (1897) The Amblyopsidae, the blind fish of America. Rep Brit Assoc Advanc Sci 1897: 685–686.

Eigenmann CH (1905) Divergence and convergence in fishes. Biol Lect Mar Biol Lab Woods Hole 8: 59–66.

Eigenmann CH (1909) Cave Vertebrates of America. A Study in Degenerative Evolution. Carnegie Institution of Washington, Washington, D.C. 241pp.

Emerton JH (1875) Notes on spiders from caves in Kentucky, Virginia and Indiana. Amer Nat 9: 278-281.

Etnier DA, Starnes WC (1993) The Fishes of Tennessee. University of Tennessee Press, Knoxville, TN. 681 pp.

Ferguson LM (1981a) Cave Diplura of the United States, Proc 8^th^ Int Congr Speleol, Bowling Green, KY 1: 11–12.

Ferguson LM (1981b) Systematics, evolution, and zoogeography of the cavernicolous campodeids of the genus *Litocampa* (Diplura: Campodeidae) in the United States. 374 pp. Ph.D. dissertation, Virginia Polytechnic Institute and State University.

Fleming LE (1972) The evolution of the eastern North American isopods of the genus *Asellus* (Crustacea: Asellidae). Part I. Internat J Speleol 4: 221–256.

Gertsch WJ (1984) The spider family Nesticidae (Araneae) in North America, Cental America and the West Indies. Texas Mem Mus Bull 31: 1-91.

Girard CF (1859) Ichthyological notices. Proc Acad Nat Sci Phila 1859 :56–68.

Goodnight CJ, Goodnight ML (1942) New Phalangodidae (Phalangida) from the United States. Amer Mus Novitat 1188: 1-18.

Goodnight CJ, Goodnight ML (1960) Speciation among cave opilionids of the United States. Amer Midl Nat 64: 34–38.

Goricki S, Niemiller ML, Fenolio DB (2012) Salamanders. Pp. 665–676, In W.H. White and D.C. Culver (Eds.). Encyclopedia of Caves, 2^nd^ edition. Elsevier.

Hart DG, Hart CW Jr (1974) The ostracod family Entocytheridae. Acad Nat Sci Philadelphia Monogr 18: 1-239.

Hart CW Jr, Hobbs HH Jr (1961) Eight new troglobitic ostracods of the genus *Entocythere* (Crustacea, Ostracoda) from the eastern United States. Proc Acad Nat Sci Philadelphia 113: 173-185.

Hay WP (1901) Two new subterranean crustaceans from the United States. Proc Biol Soc Washington 14: 179-180.

Hay WP (1902) Observations on the crustacean fauna of Nickajack Cave, Tennessee and vicinity. Proc U.S. Nat Mus 25: 417–439.

Hedin MC (1997) Speciational history in a diverse clade of habitat-specialized spiders (Araneae: Nesticidae: Nesticus): Inferences from geographic-based sampling. Evolution 51: 1929-1945.

Hedin MC, Dellinger B (2005) Descriptions of a new species and previously unknown males of *Nesticus* (Araneae: Nesticidae) from caves in Eastern North America, with comments on species rarity. Zootaxa 904: 1-19.

Hedin MC, Thomas SM (2010) Molecular systematics of eastern North American Phalangodidae (Arachnida: Opiliones: Laniatores) demonstrating convergent morphological evolution in caves. Mol Phyl Evol 54: 107-121.

Hobbs HH Jr, Barr TC Jr (1960) Origins and affinities of the troglobitic crayfishes of North America (Decapoda: Astacidae) I. Genus *Cambarus*. Amer Midl Nat 64 :12-33.

Hobbs HH Jr, Barr TC Jr (1972) Origins and affinities of the troglobitic crayfishes of North America (Decapoda: Astacidae) II. Genus *Orconectes*. Smithson Contrib Zool 105: 1-84.

Hobbs HH Jr, Hobbs HH III, Daniel MA (1977) A review of the troglobitic decapod crustaceans of the Americas. Smithson Contrib Zool 244: 1-183.

Hoffman RL (1956) New genera and species of cavernicolous diplopods from Alabama. Geological Survey of Alabama Museum Paper 35: 5-13.

Holsinger JR (1965a) Free living mites (Acarina) in caves of the eastern United States. NSS Bull 27: 47-54.

Holsinger JR (1965b) Redescriptions of two poorly known species of cavernicolous rhagidiid mites (Acarina: Trombidiformes) from Virginia and Kentucky. Acarologia 7: 654-662.

Holsinger JR (1967) Systematics, speciation, and distribution of the subterranean amphipod genus *Stygonectes* (Gammaridae). U.S. Nat Mus Bull 259: 1-176.

Holsinger JR (1969) The systematics of the North American subterranean amphipod genus *Apocrangonyx* (Gammaridae), with remarks on ecology and zoogeography. Amer Midl Natur 81: 1-28.

Holsinger JR (1972) The freshwater amphipod crustaceans (Gammaridae) of North America. Biota of Freshwater Ecosystems, Identification Manual 5, U. S. Environmental Protection Agency, 89 pp.

Holsinger JR (1978) Systematics of the subterranean amphipod genus *Stygobromus* (Crangonyctidae), Part II: species of the eastern United States. Smithson Contrib Zool 266: 1-144.

Holsinger JR, Culver DC (1988) The invertebrate cave fauna of Virginia and a part of eastern Tennessee: zoogeography and ecology. Brimleyana 14: 1-162.

Holt PC (1963) A new branchiobdellid (Branchiobdellidae: *Cambarincola*). J Tenn Acad Sci 38: 97-100.

Holt PC (1973) Branchiobdellids (Annelida: Clitellata) from some eastern North American caves, with descriptions of new species of the genus *Cambarincola*. Internat J Speleol 5: 219-256.

Hubricht L (1943) Studies on the Nearctic fresh-water Amphipoda III. Notes on the freshwater amphipods of the eastern United States, with descriptions of ten new species. Amer Midl Natur 29: 683-712.

Hubricht L (1962) New species of *Helicodiscus* from the eastern United States. Nautilus 75: 102-107.

Hubricht L (1964) Land snails from the caves of Kentucky, Tennessee and Alabama. NSS Bull 26: 33-36.

Hubricht L (1965) Four new land snails from the southeastern United States. Nautilus 79: 4-7.

Hubricht L (1973) The land snails of Tennessee. Sterkiana 49: 11–17.

Jeannel RG (1933) Trois *Adelops* nouveaux de l’Amerique du Nord. Bull Soc Entomol France 38: 251-253.

Jeannel RG (1949) Les Coléoptères cavernicoles de la région des Appalaches. Étude systématique. Notes Biospéologiques 4: 37-104.

Jeannel R (1963) Supplement a la monographie des Anillini (1). Sur quelques especes nouvelles de l’Amerique du Nord. Revue Francaise d’Entomologie, tome 30, fasc. 3: 145-152.

Kenk R (1977) Freshwater triclads (Turbellaria) of North America. IX. The genus *Sphalloplana*. Smithson Contrib Zool 246: 1-38.

Keyserling EG (1881) Neue Spinnen aus Amerika. III. Verh K K Zool-Bot Ges Wien 31: 269-314.

Keyserling EG (1886) Die Spinnen Amerikas: Theridiidae. Volume 2, part 2. Bauer & Raspe, Nurnberg.

Klie W (1931) Campagne spéologique de C. Bolivar et R. Jeannel dans l’Amérique du Nord (1928). 3. Crustaces Ostracodes. Biospeleologica: Arch Zool Expér Gén 71: 333-344.

Koenemann S, Holsinger JR (2001) Systematics of the North American subterranean amphipod genus *Bactrurus* (Cragonyctidae). Beaufortia 51: 1-56.

Kuhajda BR, Mayden RL (2001) Status of the federally endangered Alabama cavefish, *Speoplatyrhinus poulsoni* (Amblyopsidae), in Key Cave and surrounding caves, Alabama. Environ Biol Fishes 62: 215–222.

Lazell JD, Brandon RA (1962) A new stygian salamander from the southern Cumberland Plateau. Copeia 1962: 300-306.

Lewis JJ (1982) Systematics of the troglobitic *Caecidotea* (Crustacea: Isopoda: Asellidae) of the southern Interior Low Plateaus. Brimleyana 8: 65-74.

Lewis JJ (1988) The systematics, zoogeography and life history of the troglobitic isopods of the interior plateaus of the eastern United States. Ph.D. dissertation. University of Louisville, Louisville, KY. 281 pp.

Lewis JJ (2001) A biological reconnaissance of the Rumbling Falls Cave system, Van Buren County, Tennessee. Final Report. 23 pp.

Lewis JJ (2002) *Chaetaspis aleyorum*, a new species of milliped from Tumbling Creek Cave, Missouri, with a synopsis of the cavernicolous species of *Chaetapsis* (Diplopoda: Polydesmida). Myriapodologica 7: 101-111.

Lewis JJ (2004) A biological reconnaissance of caves of the northern Cumberlands project area (Fentress, Pickett & Overton counties, Tennessee). Technical report. Tennessee Chapter of The Nature Conservancy, Nashville, TN. 63 pp.

Lewis JJ (2005a) Southern Cumberland Plateau Cave Survey, Final Report. The Nature Conservancy. 155 pp.

Lewis JJ (2005b) Six new species of *Pseudotremia* from caves of the Tennessee Cumberland Plateau (Diplopoda: Chordeumatidae: Cleidogoniidae). Zootaxa 1080:17-31.

Lewis JJ (2009a) On the identity of *Caecidotea nickajackensis* (Crustacea: Isopoda: Asellidae). Proc Biol Soc Wash 122: 215-224.

Lewis JJ (2009b) Eight new cavernicolous species of milliped genus *Pseudotremia* (Diplopoda: Chordeumatida: Cleidogonidae). Pp. 171-186 in S. M. Roble and J. C. Mitchell (eds.) A Lifetime of Contributions to Myriapodology and the Natural History of Virginia: A Festschrift in Honor of Richard L. Hoffman’s 80^th^ Birthday. Virginia Museum of Natural History Special Publication No. 16, Martinsville, Virginia.

Lewis JJ, Bowman TE (1981) The subterranean asellids (*Caecidotea*) of Illinois (Crustacea: Isopoda: Asellidae). Smithson Contrib Zool 335: 1-66.

Lewis JJ, Lewis SL (2005) Inventory of the subterranean fauna of Tims Ford State Park, Franklin County, Tennessee. Tennessee Natural Heritage Program, Nashville, TN. 31 pp.

Lewis JJ, Lewis SL (2007) A biological reconnaissance of selected caves in the Highland Rim area of central Tennessee. Technical report. Tennessee Chapter of The Nature Conservancy, Nashville, TN. 43 pp.

Lewis JJ, Lewis SL (2009) Range extension of the groundwater ostracod *Pseudocandona jeanneli* (Crustacea: Ostracoda: Candonidae). Speleobiology Notes 1: 14-16.

Lewis JJ, Reid JW (2007) Patterns and processes of groundwater invasion by copepods in the Interior Low Plateaus of the United States. Acta Carsologica 36: 279-289.

Lewis JJ, Whitaker O, Krantz GW (2010) A biological reconnaissance of the invertebrate fauna of twelve Tennessee caves with notes of the guanophilic mites of the genus *Macrocheles*. J Tenn Acad Sci 85: 53-61.

Loomis HF (1939) The millipeds collected in Appalachian caves by Mr. Kenneth Dearolf. Bull Mus Comp Zool Harvard 86: 165-193.

Loomis HF (1943) New cave and epigean millipeds of the United States, with notes on established species. Bull Mus Comp Zool Harvard 92: 373-410.

Magniez G (1981) Experimental breeding of the U.S. cavernicolous crustacean *Caecidotea recurvata* (Steeves, 1963). Proceedings of the 8^th^ International Congress of Speleology 1: 241-242.

Malcolm DR, Chamberlin JC (1960) The pseudoscorpion genus *Chitrella* (Chelonethida Syarinidae). Amer Mus Novit 1989: 1-19.

Malcolm DR, Chamberlin JC (1961) The pseudoscorpion genus *Kleptochthonius* Chamberlin (Chelonethida, Chthoniidae). Amer Mus Nov 2063: 1-35.

Marshall SA, Peck SB (1984) Distribution of cave-dwelling Sphaeroceridae (Diptera) of eastern North America. Proc Ent Soc Ontario 115: 37-41.

Martof BS, Rose FL (1962) The comparative osteology of the anterior elements of the salamanders *Gyrinophilus* and *Pseudotriton*. Copeia 1962: 727–732.

Mays JD (2002) A systematic approach to sampling the arthropod assemblage of Gregorys Cave, Great Smoky Mountains National Park. Masters thesis, Western Carolina University.

McCrady E (1954) A new species of Gyrinophilus (Plethodontidae) from Tennessee caves. Copeia 1954: 200-206.

McRitchie RG (1959) The Kenkiidae of the Nashville Area. 43 pages, 10 plates. M.A. thesis, Vanderbilt University.

Miller JA (2005) Cave adaptation in the spider genus *Anthrobia* (Araneae, Linyphiidae, Erigoninae). Zoological Scripta 34: 565-592.

Miller BT (1995) Geographic distribution. *Gyrinophilus palleucus*. Herpetol Rev 26: 103.

Miller BT, Niemiller ML (2005a) Distribution, demography, and phylogenetics of the Tennessee cave salamander complex. Technical report. Tennessee Wildlife Resources Agency, Nashville, TN. 73 pp.

Miller BT, Niemiller ML (2005b) The Tennessee cave salamander complex. Pp. 91–94 In Brown JS, Simon SS (Eds.). 2005 National Speleological Society Convention Guidebook. National Speleological Society, Huntsville, AL.

Miller BT, Niemiller ML (2007) Distribution and phylogenetics of the Tennessee cave salamander complex (*Gyrinophilus p. palleucus*, *G. p. necturoides*, and *G. gulolineatus*). Technical report. Tennessee Wildlife Resources Agency, Nashville, TN. 45 pp.

Miller BT, Niemiller ML (2008) Distribution and relative abundance of Tennessee Cave Salamanders (*Gyrinophilus palleucus* and *Gyrinophilus gulolineatus*) with an emphasis on Tennessee populations. Herpetological Conservation and Biology 3: 1–20.

Miller BT, Niemiller ML (2011) Tennessee cave salamander. *Gyrinophilus palleucus*. Pp. 175–178 In Niemiller ML, Reynolds RG (Eds.). The Amphibians of Tennessee. University of Tennessee Press, Knoxville, TN.

Miller BT, Walther L (1994) Geographic distribution. *Gyrinophilus palleucus*. Herpetol Rev 25: 73.

Mills HB (1948) New North American Tomocerinae. Annals of the Entomological Society of America 41: 353-359.

Muchmore WB (1965) North American cave pseudoscorpions of the genus *Kleptochthonius*, subgenus *Chamberlinochthonius* (Chelonethida, Chthoniidae). Amer Mus Novit 2234: 1-27.

Muchmore WB (1966) Two new species of *Kleptochthonius* (Arachnida, Chelonethida) from a cave in Tennessee. J Tenn Acad Sci 41: 68-69.

Muchmore WB (1966) A new cavernicolous pseudoscorpion of the genus *Microcreagris* from southern Tennessee. Ent News 77: 97-100.

Muchmore WB (1970) A new troglobitic trichoniscid isopod of the genus *Caucasonethes*. J Tenn Acad Sci 45: 27-28.

Muchmore WB (1974) New cavernicolous species of *Kleptochthonius* from Virginia and West Virginia. Ent News 85: 81–84.

Muchmore WB (1976) New cavernicolous species of *Kleptochthonius* and recognition of a new species group within the genus (Pseudoscorpionida: Chthoniidae). Ent News 87: 211-217.

Muchmore WB (1996) The genus *Tyrannochthonius* in the eastern United States (Pseudoscorpionida: Chthoniidae). Part II. More recently discovered species. Insecta Mundi 10: 153-168.

Nicholas G (1960) Checklist of macroscopic troglobitic organisms of the United States. Amer Midl Nat 64: 123–160.

Niemiller ML (2004) Geographic distribution. *Gyrinophilus porphyriticus*. Herpetol Rev 35: 76.

Niemiller ML (2005) The herpetofauna of the upper Duck River watershed in Coffee County, Tennessee. J Tenn Acad Sci 80: 6–12.

Niemiller ML (2006) Systematics of the Tennessee cave salamander complex (Gyrinophilus palleucus) in Tennessee. M.S. Thesis. Middle Tennessee State University, Murfreesboro, TN.

Niemiller ML (2011) Evolution, speciation, and conservation of amblyopsid cavefishes. Ph.D. dissertation, University of Tennessee, Knoxville.

Niemiller ML, Fitzpatrick BM (2008) Phylogenetics of the southern cavefish (*Typhlichthys subterraneus*): implications for conservation and management. Proc Nat Cave Karst Managmt Symp, St. Louis, MO 18: 79–88.

Niemiller ML, Fitzpatrick BM, Miller BT (2008) Recent divergence-with-gene-flow in Tennessee cave salamanders (Plethodontidae: *Gyrinophilus*) inferred from gene genealogies. Molecular Ecology 17: 2258–2275.

Niemiller ML, Fitzpatrick BM, Shah P, Schmitz L, Near TJ. In press. Evidence for repeated loss of selective constraint in rhodopsin of amblyopsid cavefishes (Teleostei: Amblyopsidae). Evolution.

Niemiller ML, Miller BT (2005) Common salamanders of Tennessee caves. Tenn Caver 2: 12–18.

Niemiller ML, Miller BT (2009) A survey of the cave-associated amphibians of the eastern United States with an emphasis on salamanders. Proc Int Congr Speleol, Kerrville, TX 15: 249–256.

Niemiller ML, Miller BT (2010) *Gyrinophilus gulolineatus*. Cat Amer Amphibians Reptiles 862: 1–4.

Niemiller ML, Miller BT (2011) Berry cave salamander. *Gyrinophlus gulolineatus*. Pp. 172–174 In Niemiller ML, Reynolds RG (Eds.). The Amphibians of Tennessee. University of Tennessee Press, Knoxville, TN.

Niemiller ML, Miller BT, Fitzpatrick BM (2009) Systematics and evolutionary history of subterranean salamanders of the genus *Gyrinophilus*. Proc Int Congr Speleol, Kerrville, TX 15: 242–248.

Niemiller ML, Miller BT, Fitzpatrick BM (2010a) Review of the scientific literature and research for the USFWS review for potential listing of the Berry Cave salamander (*Gyrinophilus gulolineatus*). Technical Report. U.S. Fish and Wildlife Service, Cookeville, TN. 22 pp.

Niemiller ML, Miller BT, Fitzpatrick BM (2010b) Status and distribution of the amblyopsid fishes *Forbesichthys agassizii* and *Typhlichthys subterraneus* in Tennessee. Technical report. Tennessee Wildlife Resources Agency, Nashville, TN. 70 pp.

Niemiller ML, Near TJ, Fitzpatrick BM (2012) Delimiting species using multilocus data: diagnosing cryptic diversity in the southern cavefish *Typhlichthys subterraneus* (Teleostei: Amblyopsidae). Evolution 66: 846-866.

Niemiller ML, Poulson TL (2010) Studies of the Amblyopsidae: past, present, and future. Pp. 169–280 In Trajano E, Bichuette ME, Kappor BG (Eds.). The Biology of Subterranean Fishes. Science Publishers, Enfield, NH.

Niemiller ML, Reynolds RG, Glorioso BM, Spiess J, Miller BT (2011) Herpetofauna of the cedar glades and associated habitats of the Inner Central Basin of middle Tennessee. Herpetol Conserv Biol 6: 135–149.

Nolfi DC (2009) Review and assessment of known cavernicoles and rare epigean biology of karst and caves, Great Smoky Mountains National Park. Proc Int Congr Speleol, Kerrville, TX 15: 1323–1328.

Packard AS (1871) The Mammoth Cave and its inhabitants. On the Crustaceans and insects. Am Nat 5: 744-761.

Packard AS (1879) Zoology for Students and General Readers*.* New York: Henry Holt and Co. pp. 719.

Packard AS (1888) The cave fauna of North America, with remarks on the anatomy of the brain and origin of the blind species. Mem Nat Acad Sci 4: 1-156.

Paquin P, DuPerre N, Buckle DJ, Lewis JJ (2009) *Oreonetides beattyi*, a new troglobitic spider (Araneae: Linyphiidae) from eastern North America, and re-description of *Oreonetides flavus*. J Cave and Karst Studies 71: 2-15.

Park O (1951) Cavernicolous pselaphid beetles of Alabama and Tennessee, with observations on the taxonomy of the family. Geol Surv Alabama Mus Pap 31: 1-107.

Park O (1956) New or little known species of pselaphid beetles from southeastern United States. J Tenn Acad Sci 31: 54-100.

Park O (1958) New or little known species of pselaphid beetles, chiefly from southeastern United States. J Tenn Acad Sci 33: 39-74.

Park O (1960) Cavernicolous pselaphid beetles of the United States. Amer Midl Natur 64: 66-104.

Park O (1965) Revision of the genus *Batriasymmodes* (Coleoptera: Pselaphidae). Trans Am Micros Soc 84: 184-201.

Peck SB (1973) A systematic revision and the evolutionarybiology of the *Ptomaphagus* (*Adelops*) beetles of North America (Coleoptera; Leiodidae; Catopinae), with emphasis on cave-inhabiting species. Mus Comp Zool Bull 145: 29-162.

Peck SB (1975) The allopatric distribution of the cavernicolous beetles *Ptomaphagus hubrichti* and *Ptomaphagus barri* in Tennessee (Leiodidae: Catopinae). Ann Speleol 30: 467-470.

Peck SB (1984) The distribution and evolution of cavernicolous *Ptomaphagus* beetles in the southeastern United States with new species and records. Can J Zool 62: 730-740.

Peck SB (1989) The cave fauna of Alabama: Part I. The terrestrial invertebrates (excluding insects). Bull Nat Speleol Soc 51: 11-33.

Peck SB (1995) The cave fauna of Alabama. Part II: The insects. Bull Nat Speleol Soc 57: 1-19.

Petrunkevitch A (1925) Descriptions of new or inadequately known American spiders. Ann. Entomol. Soc. America 18: 313-323, pl. XX.

Platnick NI (1999) A revision of the Appalachian spider genus *Liocranoides* (Araneae: Tengellidae). Am Mus Novitat 3285: 1-13.

Poulson TL (1961) Cave adaptation in amblyopsid fishes. Ph.D Dissertation, University of Michigan, Ann Arbor. University Microfilms 61-2787.

Poulson TL (1963) Cave adaptation in amblyopsid fishes. American Midland Naturalist 70: 257–290.

Proudlove GS (2001) The conservation status of hypogean fishes. Environmental Biology of Fishes 62: 201–213.

Proudlove GS (2006) Subterranean Fishes of the World. International Society for Subterranean Biology, Moulis, France. 300 pp.

Redmond WH, Scott AF (1996) Atlas of Amphibians in Tennessee. Miscellaneous Publication 12. Center for Field Biology, Austin Peay State University, Clarksville, TN. 94 pp.

Reeves WK (2000) Invertebrate cavernicoles of the Great Smoky Mountains National Park, USA. The Journal of the Elisha Mitchell Scientific Society 116: 334-343.

Reid JW (1988) Copepoda (Crustacea) from a seasonal­ly flooded marsh in Rock Creek Stream Valley Park, Maryland. Proceedings of the Biological Society of Washington 101: 31-38.

Reid JW (2004) New records and new species of the genus *Diacyclops* (Crustacea: Copepoda) from subterranean habitats in southern Indiana, USA. Jeffersoniana 12: 1-65.

Rhoades R (1941) Notes on some crayfishes from Alabama caves, with the description of a new subspecies. Proc U. S. Nat Mus 91: 141-148.

Salmon JT (1964) An index to the Collembola. Bull Royal Soc New Zealand 7: 1-651.

Samoray ST, Garland HR (2002) Geographic distribution. *Gyrinophilus palleucus*. Herpetol Rev 33: 316.

Schultz GA (1970) Descriptions of new subspecies of *Ligidium elrodii* (Packard) comb. Nov. with notes on other isopod crustaceans from caves in North America (Oniscoidea). Am Midl Nat 84: 36-45.

Shear WA (1972) Studies in the milliped order Chordeumida (Diplopoda): a revision of the family Cleidogonidae and a reclassification of the order Chordeumida in the New World. Bull Mus Comp Zool 144: 151-352.

Shear WA (2010) The milliped family Trichopetalidae, Part 2: The genera *Trichopetalum, Zygonopus* and *Scoterpes* (Diplopoda: Chordeumatida, Cleidogonoidea). Zootaxa 2385: 1-62.

Simmons DD (1975) The evolutionary ecology of *Gyrinophilus palleucus*. M.S. Thesis. University of Florida, Gainsville, FL. 210 pp.

Simmons DD (1976a) A naturally metamorphosed *Gyrinophilus palleucus* (Amphibia, Urodela, Plethodontidae). J Herpetol 3: 255–257.

Simmons DD (1976b) Tennessee’s endangered cave salamander. Tenn Conservationist 42: 20–21.

Snowman CV, Zigler KS, Hedin M (2010) Caves as islands: mitochondrial phylogeography of the cave-obligate spider species *Nesticus barri* (Araneae: Nesticidae). J Arachnol 38: 49–56.

Sokolov IM, Carlton C, Cornell JF (2004) Review of *Anillinus*, with descriptions of 17 new species and a key to soil and littler species (Coleoptera: Carabidae: Trechinae: Bembidiini). Coleopt Bull 58: 185-233.

Steeves HR III (1963) The troglobitic asellids of the United States: the *Stygius* Group. Amer Midl Natur 69: 470-481.

Steeves, HR III (1966) Evolutionary aspects of the troglobitic asellids of the United States: the *hobbsi*, *stygius* and *cannulus* groups. Amer Midl Nat 75: 392-403.

Steeves, HR III, Holsinger JR (1968) Biology of three new species of troglobitic asellids from Tennessee. Amer Midl Nat 80: 75-83.

Stout VR (1911) A new subterranean freshwater amphipod. Pomona Coll Journ Entomol 3: 569-571.

Swofford DL (1982) Genetic variability, population differentiation, and biochemical relationships in the family Amblyopsidae. Master’s Thesis, Eastern Kentucky University, Richmond, KY. 386 pp.

Tellkampf T (1844) Beschreibung einiger neuer in der Mammuth-Höhle in Kentucky aufgefundener Gattungen von Gliedertieren. Archiv fur Naturgeschichte 10: 318–322.

Valentine JM (1931) New cavernicole Carabidae of the subfamily Trechinae Jeannel. Journal of the Elisha Mitchell Science Society 46: 247-258.

Valentine JM (1932) A classification of the genus *Pseudanophthalmus* Jeannel (fam. Carabidae) with descriptions of new species and notes on distribution. Journal of the Elisha Mitchell Science Society 48: 261-280.

Valentine JM (1937) Anophthalmid beetles (fam. Carabidae) from Tennessee caves. Journal of the Elisha Mitchell Science Society 53: 93-100.

Valentine JM (1945) Speciation and raciation in *Pseudanophthalmus* (cavernicolous Carabidae). Trans Conn Acad Arts and Sci 36: 631-72.

Valentine JM (1948) New anophthalmid beetles from the Appalachian region. Geol Surv Alabama Mus Pap 27: 1-20.

Valentine JM (1952) New genera of anophthalmid beetles from Cumberland caves (Carabidae, Trechinae). Geol Surv Alabama Mus Pap 34.

Vandel A (1965) Les Trichoniscidae cavernicoles (Isopoda terrestria; Crustacea) de l’Amerique du Nord. Ann Spéléol 20: 347-389.

Wakefield KR, Zigler KS (2012) Obligate subterranean fauna of Carter State Natural Area, Franklin County, Tennessee. Speleobiol Notes 4: 24–28.

Wallace RL (1984) Biological survey report of the Great Smoky Mountains National Park caves. Technical report. National Park Service, Gatlinburg, TN.

Wallace RL (1989) Biological survey report of the Great Smoky Mountains National Park caves. Technical report. National Park Service, Gatlinburg, TN.

Wallace RL (1990) Myhr Cave fauna. Technical report. National Park Service, Gatlinburg, TN.

Woods LP, Inger RF (1957) The cave, spring and swamp fishes of the family Amblyopsidae of central and eastern United States. Amer Midl Nat 58: 232-256.

Yeatman HC (1964) A new cavernicolous cyclopoid copepod from Tennessee and Illinois. J Tenn Acad Sci 39: 95-98.

Yeatman HC (1967) Artificially metamorphosed neotenic salamanders. J Tenn Acad Sci 42: 16–22.

Yeatman HC, Miller HB (1985) A naturally metamorphosed *Gyrinophilus palleucus* from the type-locality. J Herpetol 19: 304–306.

Zhang J, Holsinger JR (2003) Systematics of the freshwater amphipod genus *Crangonyx* (Crangonyctidae) in North America. Virginia Mus Nat Hist Mem 6: 1–274.
